# Age-related changes in brain phospholipids and bioactive lipids in the APP knock-in mouse model of Alzheimer’s disease

**DOI:** 10.1186/s40478-021-01216-4

**Published:** 2021-06-29

**Authors:** Ceren Emre, Khanh V. Do, Bokkyoo Jun, Erik Hjorth, Silvia Gómez Alcalde, Marie-Audrey I. Kautzmann, William C. Gordon, Per Nilsson, Nicolas G. Bazan, Marianne Schultzberg

**Affiliations:** 1 Department of Neurobiology, Care Sciences and Society, Division of Neurogeriatrics, Center for Alzheimer Research, Karolinska Institutet, Stockholm, Sweden; 2 Neuroscience Center of Excellence, School of Medicine, Louisiana State University Health New Orleans, 2020 Gravier Street, Suite D, New Orleans, LA 70112 USA

**Keywords:** Alzheimer, Amyloid, Astrocyte, Inflammation, Lipid mediators, Microglia, Phospholipids, Resolution

## Abstract

Sustained brain chronic inflammation in Alzheimer’s disease (AD) includes glial cell activation, an increase in cytokines and chemokines, and lipid mediators (LMs), concomitant with decreased pro-homeostatic mediators. The inflammatory response at the onset of pathology engages activation of pro-resolving, pro-homeostatic LMs followed by a gradual decrease. We used an APP knock-in (*App* KI) AD mouse that accumulates β-amyloid (Aβ) and presents cognitive deficits (at 2 and 6 months of age, respectively) to investigate LMs, their precursors, biosynthetic enzymes and receptors, glial activation, and inflammatory proteins in the cerebral cortex and hippocampus at 2-, 4-, 8- and 18-month-old in comparison with wild-type (WT) mice. We used LC-mass-spectrometry and MALDI molecular imaging to analyze LMs and phospholipids, and immunochemistry for proteins. Our results revealed an age-specific lipid and cytokine profile, and glial activation in the *App* KI mice. Despite an early onset of Aβ pathology, pro-inflammatory and pro-resolving LMs were prominently increased only in the oldest age group. Furthermore, the LM biosynthetic enzymes increased, and their receptor expression decreased in the aged *App* KI mice. Arachidonic acid (AA)-containing phospholipid molecular species were elevated, correlating with decreased cPLA2 activity. MALDI molecular imaging depicted differential distribution of phospholipids according to genotype in hippocampal layers. Brain histology disclosed increased microglia proliferation starting from young age in the *App* KI mice, while astrocyte numbers were enhanced in older ages. Our results demonstrate that the brain lipidome is modified preferentially during aging as compared to amyloid pathology in the model studied here. However, alterations in phospholipids signal early pathological changes in membrane composition.

**Supplementary Information:**

The online version contains supplementary material available at 10.1186/s40478-021-01216-4.

## Introduction

Alzheimer’s disease (AD) is a progressive neurodegenerative disease and the most common form of dementia, accounting for 60–80% of dementia patients [30]. The major risk factor for AD is age, although the exact cause of the disease and pathogenic mechanisms are still unknown. The neuropathological hallmarks of AD are an accumulation of extracellular β-amyloid (Aβ), hyperphosphorylation of tau filaments and neuroinflammation. Neuroinflammation, an early event of AD pathology [54–56], is triggered by several events including glial cells activated by Aβ plaques, that release pro-inflammatory factors causing neuronal damage. Neuroinflammation is a dynamic process that becomes more complex and damaging during disease progression. However, a rising level of neuroinflammation also takes place in healthy aging (See [25]). Therefore, defining molecular markers and signaling cascades that distinguish the pathological neuroinflammation in AD at early stages would be instructive to understand disease onset and to identify therapeutic targets. The inflammatory response sustains homeostasis by active resolution of inflammation that contributes to clearance of immune cells and cell debris as well as to tissue repair [51, 76]. These actions are in part governed by lipid mediator (LM) synthesis switching from pro-inflammatory to a pro-resolving profile [44] and failure of this switching is associated with chronic inflammation in AD [33, 49, 99, 107]. Resolution of inflammation is set in motion as an active process that counteracts polymorphonuclear neutrophil (PMN) infiltration and downregulates the production of pro-inflammatory cytokines. At the same time, anti-inflammatory cytokine expression is up-regulated and non-phlogistic phagocytosis modulated [77, 85]. Impaired resolution of inflammation has been documented in *post mortem* AD brains that display marked alterations in pro-resolving LMs and their receptors [49, 99, 109].

Cell membrane phospholipids store bioactive LM precursors and are thus key players in signal transduction, homeostasis and brain function. Aging modifies brain lipid composition [81] and is associated with functional decline. At early 20's, the brain begins to lose phospholipids, reaching 10–20% loss at the age of 80 to 90 for healthy individuals [88]. Studies on AD brains showed alterations in lipid metabolism and pathways [21, 67]. Membrane structure and function are disturbed when phospholipid composition changes, resulting in synaptic loss and contributing to AD pathology [31]. Brain phospholipids show decreased content of phosphatidylethanolamine (PE) and phosphatidylinositol (PI) [69, 102] in AD *post mortem* brains compared to controls, while phosphatidylcholine (PC) levels were decreased [27] or unchanged [102]. Studies on fatty acid composition, however, demonstrated a progressive decline with age particularly of the omega-3 fatty acid docosahexaenoic acid (DHA) in AD brains [3, 16]. Decreased pro-resolving LMs and elevated pro-inflammatory LMs have been shown in both human CSF and *post mortem* brains of AD patients [99]. Although there is evidence of lipid dysfunctions in AD, it is still unclear how the acyl chains of phospholipids, as precursors of bioactive LMs are affected at different stages of pathology in the AD brain and during normal aging. Increased levels of the LM receptors BLT1, ChemR23 and LXA4R [20, 99] have been demonstrated in human *post-mortem* AD brains. However, changes in their expression during normal aging or during the progression of AD are not known.

To address these questions, we used an *App* knock-in AD mouse model, *App*^*NL−G−F/NL−G−F*^ which carries the Swedish, Iberian, and Artic mutations to induce high expression of Aβ_42_. This model expresses amyloid precursor protein (APP) with endogenous levels and Aβ pathology beginning at 2 months of age with cognitive deficits observed at 6–8 months of age [73]. The goal of this study was to assess phospholipid molecular species and inflammatory markers during aging and in the *App*^*NL−G−F/NL−G−F*^ mice of 2-, 4-, 8- and 18-months age. The data were analyzed by a uni- as well as a multivariate approach, the latter offering an overview of the factors discriminating between WT and *App*^*NL−G−F/NL−G−F*^ mice of different ages, as well as complementing the univariate comparisons with an analysis more suitable for a large number of factors.

This study aims to pinpoint at which stage in disease progression lipid metabolism and signaling of resolution of inflammation are changing, since they represent a promising therapeutic strategy for AD by stimulating phagocytic removal of Aβ_42_ and by decreasing inflammation [101, 109], and protecting neurons ([109].

## Materials and methods

### Antibodies and reagents

All primary and secondary antibodies and reagents used in this study are listed in Table 1.

**Table 1:** Primary and secondary antibodies and reagents

Protein targeted	Host	Dilution	Provider	Catalogue number
*Primary antibodies*				
Akt	Rabbit	1:500	Cell Signaling	4691S
BLT1	Rabbit	1:400	Cayman	120114
ChemR23	Mouse	1:500	Santa Cruz	SC-398769
COX-1	Rabbit	1:400	R&D Systems	4841S
c-PLA2	Rabbit	1:400	Abcam	Ab73406
ERK1/2	Rabbit	1:500	Cell Signaling	4696S
FPR2	Rabbit	1:500	Santa Cruz	Sc-66901
Galectin-3	Goat	1:200	R&D Systems	AF1197
GFAP	Rabbit	1:800	Dako	Z033401-2
GPR18	Rabbit	1:400	Sigma-Aldrich	SAB4501252
Iba1	Rabbit	1:400	Wako	019-19741
JUNK	Rabbit	1:500	Cell Signaling	9252S
LGR6	Rabbit	1:500	Invitrogen	PA5-102099
MCSFR1	Mouse	1:500	R&D Systems	MAB3291
PLA1	Rabbit	1:500	LSBio	LS-1310189
p38	Rabbit	1:500	Cell Signaling	9212S
p-Akt	Mouse	1:400	Cell Signaling	12694S
p-ERK1/2	Mouse	1:500	Cell Signaling	9106S
p-JUNK	Mouse	1:500	Cell Signaling	9255S
p-cPLA2(S505)	Rabbit	1:200	Cell Signaling	2831S
p-p38	Mouse	1:200	Cell Signaling	9216S
p-5-LOX	Rabbit	1:400	R&D Systems	pp2001
S100β	Mouse	1:400	Sigma-Aldrich	AMAB91038
TMEM119	Rabbit	1:400	Thermo Fisher	PA5-62505
Trem-2	Sheep	1:400	R&D Systems	AF1729
YKL-40	Rat	1:500	R&D Systems	MAB2649
5-LOX	Rabbit	1:500	Abcam	Ab169755
15-LOX	Rabbit	1:400	Hans-Erik Claesson, KI	Ab154
Aβ(1–16) peptide	Mouse	1:500	BioLegend	803001
*Secondary antibodies and reagents*				
Alexa Fluor Plus 594 anti-mouse	Donkey	1:400	Invitrogen	A32744
Alexa Fluor Plus 488 anti-rabbit	Donkey	1:400	Invitrogen	A32790
IRDye®800CW anti-rabbit	Donkey	1:15,000	LiCor	926-32213
IRDye®680RD anti-mouse	Donkey	1:15,000	LiCor	926-68072
IRDye®800CW anti-rat	Goat	1:15,000	LiCor	926-32219
IRDye®800CW anti-goat	Donkey	1:15,000	LiCor	926-32214
Intercept® TBS Blocking buffer			LiCor	927-66003

### Animals

**Fig. 1:**
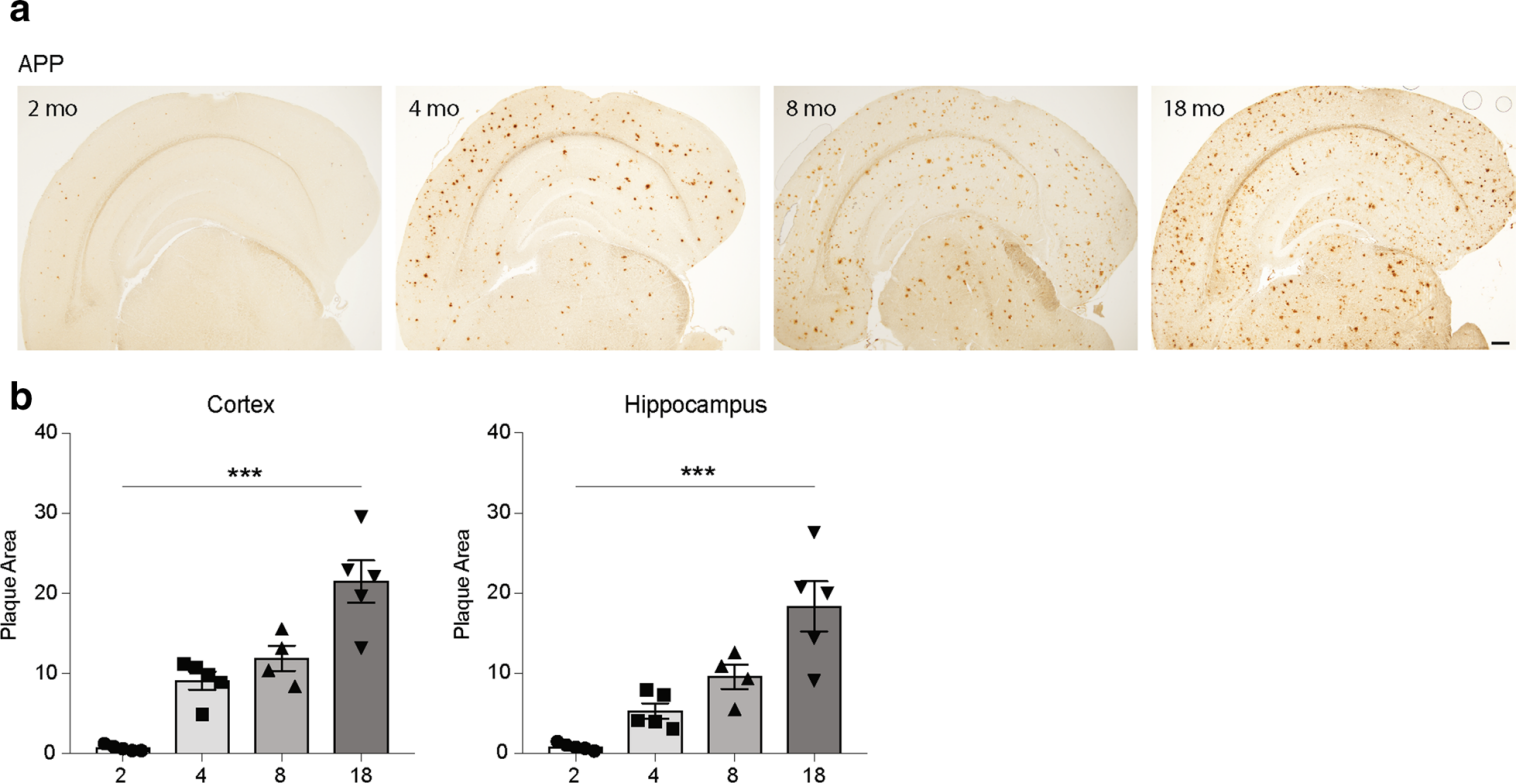
** Age-dependent β amyloid (Aβ) plaque deposition in the App KI mouse brain**. **a** Immunohistochemistry for Aβ with the 6E10 antibody in coronal sections of brains from *App* KI mice of 2-, 4-, 8- and 18-months age. **b** Densitometry of the area covered by 6E10-positive plaques in *App* KI mouse brain was measured using Image J. Kruskal–Wallis one-way analysis of variance test with Dunn’s post hoc test (*P < 0.05, **P < 0.01, ***P < 0.001) was used for multiple comparisons between ages. *App* KI = amyloid precursor protein knock-in (*App*^*NL−G−F/NL−G−F*^). Scale bar = 300 μm

All studies involving mice were performed according to the guidelines of Comparative Medicine (KM-B, Karolinska Institutet) and the Stockholm ethical committee for animal experiments (6-14, 1433-2018, 12370–2019). Young and aged male C57BL/6 J mice were purchased from The Jackson Laboratory (Bar Harbor, ME, USA). The *App*^*NL−G−F/NL−G−F*^ mice (referred here as *App* KI) were bred in the Karolinska Institutet animal facility. WT and *App* KI mice of 2, 4, 8 and 18 months of age were used in the studies. Aβ pathology was visualized by immunohistochemistry using antibodies raised against Aβ peptide (6E10) (Fig. 1). The mice were housed (5/cage) and maintained under pathogen-free conditions on a 12 h light/dark cycle with free access to water and food.

The mice were anaesthetized with isoflurane and intracardially perfused with 0.9% physiological saline prior to harvesting the brains. The right hemisphere was fixed with 4% paraformaldehyde (PFA) in 0.1 M phosphate buffer for 48 h, soaked in 30% sucrose in phosphate buffer, and subsequently embedded in paraffin for immunohistochemistry. Hippocampus and cerebral cortex were dissected from the left hemisphere, frozen in dry ice, and stored at − 80 °C for Western blot analysis, Meso Scale analysis and LC–MS/MS analysis. Twelve mice from each age (2, 4, 8 and 18 months) were used. Additional whole brains (n = 3/group) were frozen in dry ice and stored at − 80 °C for MALDI-IMS imaging.

### Western blot

Briefly, frozen tissues were homogenized in radioimmunoprecipitation assay (RIPA) buffer (1:10 w/v) containing protease and phosphatase inhibitor cocktails (Sigma Aldrich, Stockholm, Sweden, Thermo Fisher Scientific, Gothenburg, Sweden) using a homogenizer and sonicator. The homogenates were centrifuged at 12,000 × *g* for 20 min at 4 °C and total protein levels determined using a BCA kit (Thermo Fisher Scientific, Gothenburg, Sweden). Equal amounts of total protein (20 µg) were separated by sodium dodecyl sulphate polyacrylamide gel electrophoresis (SDS-PAGE) for 1 h at 160 V using Invitrogen precast NuPAGE 4–12% 15-well Bis–Tris gels (Thermo Fisher Scientific, Gothenburg, Sweden). Then proteins were transferred to nitrocellulose membranes using a Bio-Rad wet transfer cell (Bio-Rad, Solna, Sweden). After blocking in Li-COR Tris-buffered saline (TBS)-based blocking buffer (Li-COR Biosciences, Lincoln, USA) the membranes were incubated with primary antibodies over night at 4 °C. Following washing (3 × 10 min) in TBS-0.1% Tween-20 (TBS-T), the membranes were incubated with IRDye™ secondary antibodies (Li-COR Biosciences, Lincoln, USA). After washing (3 × 10 min) in TBS-T, the membranes were imaged in the Odyssey Infrared Imaging System (Li-COR Biosciences, Lincoln, USA), and quantification of signals was performed using Image Studio 5.2.

### Immunohistochemistry

Paraffin-embedded tissue blocks were cut into 10 μm thick coronal sections, deparaffinized, and pre‐treated for antigen retrieval in citrate buffer (pH 6, 95 °C, 5 min). Then sections were blocked with 5% normal donkey or goat serum for 30 min at room temperature (RT), followed by overnight incubation at 4 °C with primary antibodies. After washing in 0.01 M phosphate‐buffered saline (PBS), pH 7.4, the immunoreactivity was detected indirectly using secondary antibodies conjugated with Alexa Fluor 488 or 594 for immunofluorescence, or with the Polyvalent HRP/DAB detection kit (Abcam, Cambridge, USA). After washing, sections were mounted and examined with a Leica epifluorescence microscope or a Nikon Eclipse E800 microscope (Bergman-Labora, Stockholm, Sweden).

### Quantification of amyloid pathology, microglia and astrocytes

The amyloid pathology was analyzed in the cerebral cortex and hippocampus by semi‐quantitative densitometry of Aβ peptide (6E10 antibody) immunoreactivity in two sections/ animal for each age (N = 4–5/age group). The analysis was performed in images captured by a Nikon Eclipse E800 microscope with a 2X objective. The area occupied by Aβ-positive plaques was measured by applying threshold using NIH Image J 1.5 software program (NIH, USA). All images were collected under the same lighting conditions and settings.

Microglia and astrocytes were analyzed in the cerebral cortex and hippocampus areas by counting cells with positive immunoreactivity to ionized calcium-binding adapter molecule 1 (Iba1), glial fibrillary acidic protein (GFAP) and S100 calcium-binding protein β (S100β), respectively (2 sections/animal, n = 5–6 animals/group). Images of Iba1-stained sections were captured with a Nikon camera using a 10X objective, and images of GFAP and S100β stained sections were captured with a Nikon camera using a 20X objective. Cells were counted using NIH ImageJ (United States National Institutes of Health) with the cell counter plug-in. Iba1-positive cells were counted in the cerebral cortex, *Cornu Ammunis 1* (CA1) and dentate gyrus (DG), each in 1 field/section. GFAP- and S100β-positive cells were counted in the cerebral cortex (2 fields/section), CA1 (2 fields/section) and DG (2 fields/section). To produce data reflecting the true number of cells in the tissue, the number of counted cells per field was normalized to the area of the field.

### Analysis of inflammatory markers by multi-immunoassays

Cytokines and chemokines were analyzed in the supernatants after tissue homogenization (see Western blot above) using 96-well V-PLEX Mouse cytokine 19-plex kit (Proinflammatory Panel 1 and Cytokine Panel-1) (#K15255D; Meso Scale, Rockville, MD, USA) according to the manufacturer's instructions. Briefly, 50 μl of the supernatants were added per well and incubated for 2 h at RT followed by washing with 0.05% Tween‐20 in 1 M PBS. The plates were then incubated with detection antibodies for 2 h at RT. Subsequently, reading buffer was added to generate an electrochemiluminescence signal, and the levels of markers were read in the Meso Quickplex SQ 120 (Meso Scale, Rockville, MD, USA) with the Discovery Workbench 4.0 software. In the case of values below the lowest detectable level, the lower limit of detection (LLOD) was assigned. Detailed information on concentrations of cytokines/chemokines detected, LLOD, and the lower limit of quantification (LLOQ) are presented in Table 2. Heatmaps were generated using the Morpheus software (https://software.broadinstitute.org/morpheus).

**Table 2:** Cytokines and chemokines detected in brain homogenates. Statistical analysis between WT and APP KI per age group

Cortex	Median (pg/ml)				Median (pg/ml)				Median (pg/ml)				Median (pg/ml)				LLOD	LLOQ
	2mo				4mo				8mo				18mo				pg/ml	pg/ml
	*P* value	WT	APP KI		*P* value	WT	APP KI		*P* value	WT	APP KI		*P* value	WT	WT	APP KI		
IL-5	0.009	0.368	0.553	↑	0.18	1.135	0.64	↓	0.999	0.301	0.301		0.002	1.075	1.422	↑	0.06	0.302
IL-4	0.03	0.338	0.461	↑	0.589	0.357	0.334	↓	0.143	0.264	0.304	↑	0.002	0.336	0.463	↑	0.11	0.818
IL-10	N/A	NA	NA		0 .818	2.034	2.098	↑	NA	NA	NA		0.065	1.849	2.23	↑	0.94	7.26
IL-27p28/1L-30	0.818	16.45	15.83	↓	0.041	6.471	4.711	↓	0.589	11.14	9.958	↓	0.041	19.26	25.98	↑	1.39	5.91
TNF-α	0.029	0.286	0.383	↑	0.394	0.833	0.685	↓	0.009	0.183	0.46	↑	0.004	0.847	2.041	↑	0.13	980
KC-GRO	0.002	3.092	5.03	↑	0.002	30.47	11.66	↓	0.026	4.811	7.914	↑	0.002	13.57	26.45	↑	0.24	3.29
IL-1β	0.065	0.434	0.533	↑	0.24	0.587	0.695	↑	0.065	0.712	1.865	↑	0.002	0.816	5.292	↑	0.11	0.72
IL-6	0.026	11.85	17.52	↑	0.041	33.73	18.88	↓	0.699	7.453	7.908	↑	0.002	29.61	39.1	↑	0.61	7.61
IL-2	0.052	0.502	0.910	↑	0.132	1.399	0.825	↓	0.143	0.491	0.588	↑	0.002	1.107	1.559	↑	0.22	1.03
IL-12p70	0.004	54.45	78.36	↑	0.31	125.1	72.88	↓	0.589	24.36	31.69	↑	0.026	106.5	129.1	↑	9.95	179
IFN-y	0.009	0.226	0.31	↑	0.121	0.086	0.045	↓	0.258	0.212	0.191	↓	0.002	0.07	0.12	↑	0.04	0.39
MIP-2	0.18	1.054	1.313	↑	0.004	2.973	1.4	↓	0.002	1.243	2.401	↑	0.002	2.395	5.52	↑	0.053	0.58
IP-10	0.093	6.532	5.619	↓	0.485	11.98	13.33	↑	0.002	6.189	33.32	↑	0.002	9.174	109.8	↑	0.328	2.15
MIP-1α	0.18	8.51	9.634	↑	0.002	6.763	61.42	↑	0.002	5.454	167.1	↑	0.002	11.03	353.3	↑	0.081	0.38
IL-15	0.818	176.4	174.2	↓	0.002	72.01	49.71	↓	0.818	176.5	165.6	↓	0.041	233.5	296.1	↑	16	43.2
IL-17A/F	0.513	6.004	6.231	↑	0.004	6.024	3.43	↓	0.485	6.001	4.217	↓	0.041	9.669	11.7	↑	0.231	1.39
MCP-1	0.132	31.495	33.37	↑	0.286	13.32	14.95	↑	0.132	27.6	21.52	↓	0.004	37.3	51.76	↑	0.672	4.42
IL-9	0.31	10.743	12.11	↑	0.009	16.28	11.99	↓	0.222	11.52	7.516	↓	0.065	16.55	19.64	↑	3.84	21.9
IL-33	0.31	251.58	285.6	↑	0.18	156.1	190.2	↑	0.18	359.4	499.1	↑	0.002	521.8	2016.4	↑	0.364	1.85

Hippocampus	Median (pg/ml)				Median (pg/ml)				Median (pg/ml)				Median (pg/ml)					
	2mo				4mo				8mo				18mo					
	*P* value	WT	APP KI		*P* value	WT	APP KI		*P* value	WT	APP KI		*P* value	WT	APP KI			
IL-5	0.18	0.389	0.468	↑	0.093	0.411	0.496	↑	0.24	0.214	0.231	↑	0.394	0.518	0.54	↑	0.06	302
IL-4	0.998	0.378	0.39	↑	0.394	0.335	0.372	↑	0.307	0.188	0.202	↑	0.784	0.366	0.36	↓	0. 11	0.818
IL-10	NA	NA	NA		NA	NA	NA		NA	NA	NA		NA	NA	NA		0.9.4	7.26
IL-27p28/1L-30	0.132	12.79	15.96	↑	0.937	12.52	13.47	↑	0.178	5.8	7.724	↑	0.041	20.38	24.69	↑	1.39	5.91
TNF-α	0.065	0.201	0.282	↑	0.084	0.258	0.319	↑	0.191	0.13	0.198	↑	0.002	0.413	1.126	↑	0.13	0.98
KC-GRO	0.18	5.067	6.519	↑	0.002	14.14	5.7	↓	0.394	3.548	5.757	↑	0.009	8.711	14.45	↑	0. 24	3.29
IL-1β	0.31	0.32	0.387	↑	0.026	0.369	0.509	↑	0.24	0.277	0.44	↑	0.002	0.851	3.053	↑	0.11	0.72
IL-6	0.165	11.87	15.05	↑	0.18	13.9	16.86	↑	0.792	5.006	5.566	↑	0.18	17.02	19.71	↑	0.61	7.61
IL-2	0.455	0.666	0.757	↑	0.699	0.614	0.659	↑	0.571	0.282	0.315	↑	0.937	0.726	0.754	↑	0.22	1.03
IL-12p70	0.394	46.42	49.82	↑	0.699	45.81	46.28	↑	0.571	9.232	11.074	↑	0.784	56.46	53.27	↓	9.95	179
IFN-y	0.288	0.241	0.253	↑	0.104	0.211	0.25	↑	0.485	0.147	0.158	↑	0.197	0.262	0.279	↑	0.04	390
MIP-2	0.31	0.861	0.942	↑	0.041	2.505	1.216	↓	0.31	0.517	0.991	↑	0.002	2.466	4.595	↑	0.053	0.58
IP-10	0.31	9.215	8.406	↓	0.699	9.973	10.34	↑	0.004	3.636	15.35	↑	0.002	12.49	88.79	↑	328	2.15
MIP-1α	0.589	3.414	3.968	↑	0.002	3.416	19.48	↑	0.002	1.928	54.22	↑	0.002	9.231	304.9	↑	0.081	0.38
IL-15	0.24	154.9	172.6	↑	0.818	190.5	166.9	↓	0.931	69.34	80.13	↑	0.026	254.8	322.1	↑	16	43.2
IL-17A/F	0.041	4.377	5.543	↑	0.31	8.042	6.996	↓	0.444	1.652	2.184	↑	0.002	9.737	13.16	↑	0.231	1.39
MCP-1	0.818	23.81	25.46	↑	0.589	27.97	26.96	↓	0.485	16.35	22.25	↑	0.015	33.78	45.04	↑	672	4.42
IL-9	0.065	7.477	11.28	↑	0.937	13.36	14.05	↑	0.485	8.438	10.31	↑	0.169	18.89	21.93	↑	3. 84	21.9
IL-33	0.24	231.3	294.7	↑	0.041	305.9	457.7	↑	0.132	294.2	457	↑	0.002	1030	2473	↑	0.364	1 _85

### LC–MS/MS analysis

LC–MS/MS analysis was performed on fresh frozen tissue specimens homogenized with CHCl_3_/MeOH (2:1). An internal standard mixture of deuterium-labelled lipids (AA-d8 (5 ng/μl), PGD2-d4 (1 ng/μl), EPA-d5 (1 ng/μl), 15-HETE-d8 (1 ng/μl), and LTB4-d4 (1 ng/μl)) was added to each sample before sonication for 30 min and storage at − 80 °C overnight. Subsequently, the samples were centrifuged at 4200 × *g* for 30 min and the supernatants collected. The pellets were washed with CHCl_3_/MeOH and centrifuged, and the supernatants from both centrifugations were combined. Two ml of distilled water, pH 3.5, were added to each supernatant, and after vortexing and centrifugation the pH of the upper phase was adjusted to 3.5–4.0 with 0.1 N HCl. The lower phase was dried down under N_2_ and then resuspended in 1 ml of MeOH. LC–MS/MS analysis was performed using a Xevo TQ UPLC (Waters, Milford, MA, USA).

Analysis of the phospholipids PC, PE, phosphatidylserine (PS) and sphingomyelin (SM) was performed in samples dried under N_2_ and resuspended in 20 μl of CH_3_CN/CHCl_3_/MeOH. Phospholipid molecular species were calculated as % of the total amount in each sample.

Analysis of fatty acids and their derivatives was performed in samples dried under N_2_ and resuspended in 1 ml MeOH. After mixing with 9 ml of H_2_O at pH 3.5, the samples were loaded onto C18 columns (Agilent, Santa Clara, CA, USA), and then eluted with methyl formate, dried under N_2_, resuspended in 50 μl MeOH/H_2_O (1:1), and injected into a column. Lipid standards (Cayman, Ann Arbor, MI, USA) were used for tuning and optimization, as well as to create calibration curves for each compound.

### MALDI-imaging

Frozen mouse brains were cut into 20 μm thick coronal sections on a cryostat and placed on coverslips to dry in a vacuum chamber over night at RT. Using thermally conductive tape, the coverslips were placed on a stainless-steel plate and covered with 2,5‐dihydroxybenzoic acid (DHB) (Fisher Scientific, Pittsburgh, PA) matrix in a sublimation chamber. MALDI-IMS was performed in a Synapt G2‐Si (Waters, Milford, MA) using a solid‐state laser (355 nm) at a firing rate of 2000 Hz for positive ion mode data collection. HD Imaging software (Waters, Milford, MA) was utilized to design the pattern of tissue scanning (15 μm spatial resolution for both horizontal and vertical movement) and for data analysis. Each image spot consisted of a collection of 1 s data acquisition. Ions created by the MALDI source were further separated by ion‐mobility‐separation with He gas in the TriWave region of the instrument, with an ion‐mobility‐separation wave velocity of 600 m/s and height of 40.0 V. The data processed with HD Imaging were converted with an in‐house program, and BioMap software (Novartis) was used to generate images. Individual MS spectra for each footprint are put together, providing a reconstructed image that shows localization of that particular phospholipid in the tissue scanned by the laser.

### Statistical analysis

Data illustrated in scatter plots with bars were presented as mean ± standard error of the mean, and data shown in scatter plots were presented with median. *P*-values less than 0.05 were considered significant. All graphs and univariate data analysis using Mann–Whitney U test and Kruskal–Wallis test followed by Dunn’s multiple comparison post hoc test were completed in GraphPad Prism version 8 (GraphPad Prism Inc., USA). In addition, a multivariate (MVA) approach with orthogonal projections to latent structures (OPLS)—discriminant analysis (DA) was used to produce models to discern group differences. OPLS and OPLS-DA were performed in Simca v15 (Umetrics, Umeå, Sweden).

## Results

### Amyloid plaque burden advances with age in *App* KI mice

In order to relate changes in lipid profiles, biosynthetic pathways and inflammatory cytokines to the development of amyloid pathology in the *App* KI mouse model, we analyzed the plaque load in mice of different ages (2-, 4-, 8- and 18-months) by measuring the area occupied by Aβ-positive plaques in the cerebral cortex and hippocampus after immunohistochemistry using an antibody to Aβ (6E10). As described previously for this mouse model [73], Aβ deposition was seen already at 2 months of age and showed a gradual increase with age (Fig. 1a, b), indicating progressive Aβ pathology.

### Pro-inflammatory and pro-resolving lipid mediators are increased with age

**Fig. 2:**
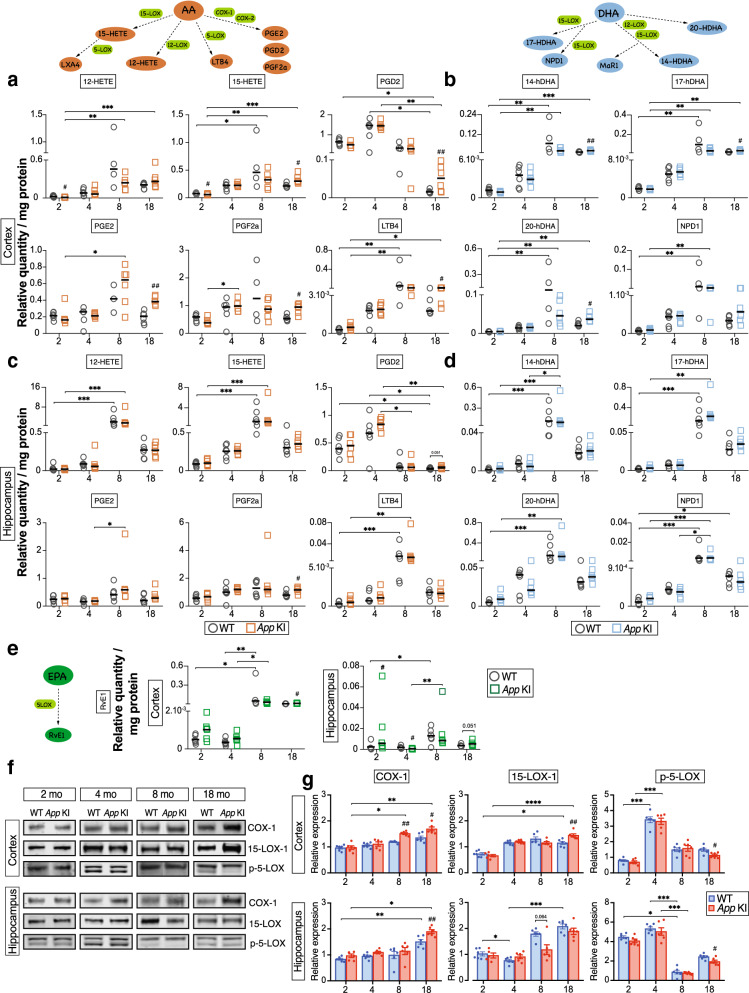
**Bioactive lipid mediators increase in cerebral cortex during developing amyloid pathology in App KI mouse model for AD**. **a**, **c** AA-, **b**, **d** DHA- and **e** EPA-derived lipid mediators (LMs) were analyzed in the cerebral cortex and hippocampus of 2, 4, 8 and 18 months-old WT (n = 4–6) and *App* KI mice (n = 6–7) using LC–MS/MS. Horizontal bars indicate median. **f** Western blots for biosynthetic enzymes (COX-1, 15-LOX-1 and p-5-LOX) and **g** corresponding densitometric analysis in cerebral cortex and hippocampus of 2, 4, 8 and 18 months-old mice (n = 6/group). Comparisons between WT (blue) and *App* KI (red) mice were performed with the Mann–Whitney U test (^#^*P* < 0.05, ^##^*P* < 0.01, ^###^*P* < 0.001). Kruskal–Wallis with Dunn’s post hoc test was used for multiple comparisons (*P < 0.05, **P < 0.01, ***P < 0.001, ****P < 0.0001) AA = arachidonic acid, DHA = docosahexaenoic acid, EPA = eicosapentaenoic acid, COX-1 = cyclooxygenase-1, 15-LOX-1 = 15-lipoxygenase-1, p-5-LOX = phosphorylated (ser523) 5-lipoxygenase

Bioactive LMs were analyzed in the cerebral cortex and hippocampus from 2-, 4-, 8- and 18-month-old *App* KI and wild-type (WT) mice to investigate their relationship to amyloid pathology. The data revealed that the most prominent changes occurred at 18 months in both *App* KI and WT mice (Fig. 2). AA-derived LMs prostaglandin E2 (PGE2), D2 (PGD2) and F2α (PGF2α), 15-hydroxyeicosatetraenoic acid (15-HETE) and leukotriene B4 (LTB_4_) were higher in the cortex of 18-month-old *App* KI compared to age-matched WT mice (Fig. 2a). In the hippocampus, on the other hand, only PGF2α was increased (Fig. 2c). At 2 months of age, however, 12-HETE and 15-HETE were lower in *App* KI mice compared to WT mice. Interestingly, lipoxin A4 (LXA_4_), also a derivative of AA and a pro-resolving LM, was higher in the cortex of 18 months old *App* KI mice than in WT mice (Additional file 1: Fig. S1A).

Similarly, the DHA-derived intermediate LMs 14-, 17- and 20-hydroxy-docosahexaenoic acid (14-HDHA, 17-HDHA and 20-HDHA) were higher in the cortex, but not in the hippocampus, of 18-month-old *App* KI mice compared to age-matched WT mice (Fig. 2b and d). The pool size of maresin 1 (MaR1), derived from DHA, was also increased (*p* = 0.065, Additional file 1: Fig. S1B) in the cortex of 18-month-old *App* KI mice, whereas neuroprotectin D1 (NDP1), was unchanged (Fig. 2b and d). Finally, resolvin E1 (RvE1), a derivative of eicosapentaenoic acid (EPA), was higher in the cortex of 18-month-old *App* KI compared to WT mice (Fig. 2e).

### Differential expression of enzymes and receptors for bioactive lipid mediators in brain regions and during aging

We investigated whether alterations in the levels of LM biosynthetic enzymes correlate with the abundance of the LMs (Fig. 2f and g). We observed that cyclooxygenase (COX)-1 and 15-lipoxygenase-1 (15-LOX-1) displayed an overall age-dependent increase in the cortex and hippocampus of both *App* KI and WT mice. In contrast, phosphorylated 5-lipoxygenase (p-5-LOX) decreased with age in both animal groups.

COX-1 levels were higher in the cortex of 8- and 18-month-old *App* KI mice, whereas in the hippocampus, the levels were higher at 4 and 18 months, compared to WT mice (Fig. 2g). The levels of 15-LOX-1 were higher in the cortex of *App* KI mice at 18 months compared to WT mice (Fig. 2g), in agreement with increased pool sizes of 15-HETE, 14- and 17-HDHA, NPD1 and MaR1 (Fig. 2a–d). Phosphorylation of Ser523 suppresses 5-LOX activity and reduces 5-LOX products [50]. We found that the levels of p-5-LOX at 18 months in *App* KI mice were lower than in WT mice in cortex and hippocampus (Fig. 2g), correlating with the elevated levels of LTB_4_ and LXA_4_ in 18-month-old *App* KI mice (Fig. 2a).

**Fig. 3:**
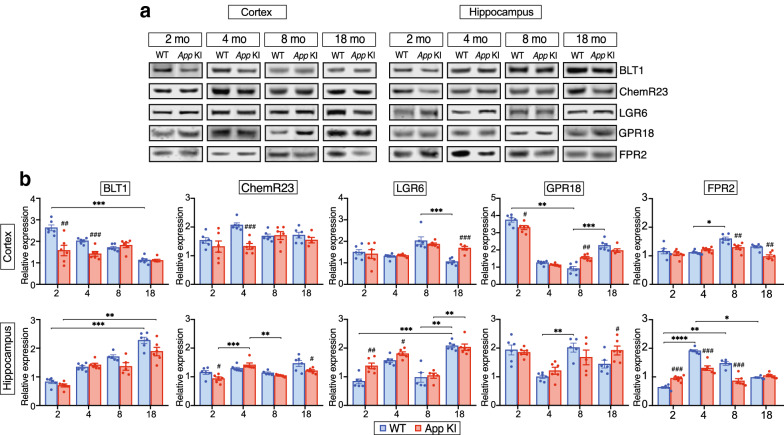
**Alterations in LM receptor levels**. The levels of BLT1, ChemR23, LGR6, GPR18 and FPR2, receptors for pro-resolving lipid mediators (LMs), in cortex and hippocampus of WT and *App* KI mice aged 2, 4, 8 and 18 months were determined by Western blot analysis. (**a**) Representative blots and (**b**) quantitative analysis are shown. Results are mean ± SEM and analysed by Mann–Whitney U test (^#^*P* < 0.05, ^##^*P* < 0.01, ^###^*P* < 0.001) for intergroup analysis. Kruskal–Wallis one-way analysis of variance test with Dunn’s post hoc test (*P < 0.05, **P < 0.01, ***P < 0.001, ****P < 0.001) was used for multiple comparisons between ages and genotypes. AA = arachidonic acid, BLT1 = leukotriene B4 receptor, ChemR23 = chemokine like receptor 1, DHA = docosahexaenoic acid, EPA = eicosapentaenoic acid, FPR2 = formyl peptide receptor 2, GPR18 = G-protein coupled receptor 18, LGR6 = leucine-rich repeat containing G-protein coupled receptor 6

We proceeded to assess the effects of aging and *App* KI pathology on multi-ligand receptors that mediate pro-resolving LM activities (Fig. 3). In the hippocampus (Fig. 3b), BLT1 (LTB_4_ receptor), targeted also by RvE1, increased with age in both *App* KI and WT mice, and similarly to leucine-rich repeat containing G protein-coupled receptor 6 (LGR6), that mediates MaR1 activities, there was a marked enhancement at 18 months. In the cortex (Fig. 3b), however, BLT1 decreased with age in the WT mice, but not in the *App* KI mice, where it remained similar through all ages. Also, the G-protein coupled receptor 18 (GPR18) showed the highest abundance at 2 months in the cortex, whereas formyl peptide receptor 2 (FPR2), that mediates LXA_4_ activities, exhibited the highest levels at 4 and 8 months in WT mice. The levels of chemokine-like receptor 1 (ChemR23) presented even levels across the age groups in both brain regions.

Therefore, the two mouse strains (Fig. 3b) revealed that the levels of BLT1 were lower in 2- and 4-month-old *App* KI compared to WT mice in the cortex but not in the hippocampus (Fig. 3b). On the other hand, ChemR23 levels were lower in the cortex of 4-month-old *App* KI compared to WT mice. In the hippocampus, however, ChemR23 was lower in 2- and 18-month-old *App* KI mice (Fig. 3b). LGR6 levels were higher in the cortex of 18-month-old *App* KI mice compared to WT mice, whereas in hippocampus the levels were higher at 2 and 4 months of age (Fig. 3b). GPR18 showed lower levels in the cortex of *App* KI mice at 2 months but higher levels at 8 months compared to WT mice, whereas in the hippocampus, increased levels were found only at 18 months of age. FPR2 was found in lower levels in the cortex of *App* KI mice at 8 and 18 months compared to WT mice. However, in the hippocampus, FPR2 levels were higher in *App* KI mice at 2 months and lower at 4 and 8 months of age compared to WT mice (Fig. 3b).

### Age-dependent changes in AA- and DHA-containing phospholipids

**Fig. 4:**
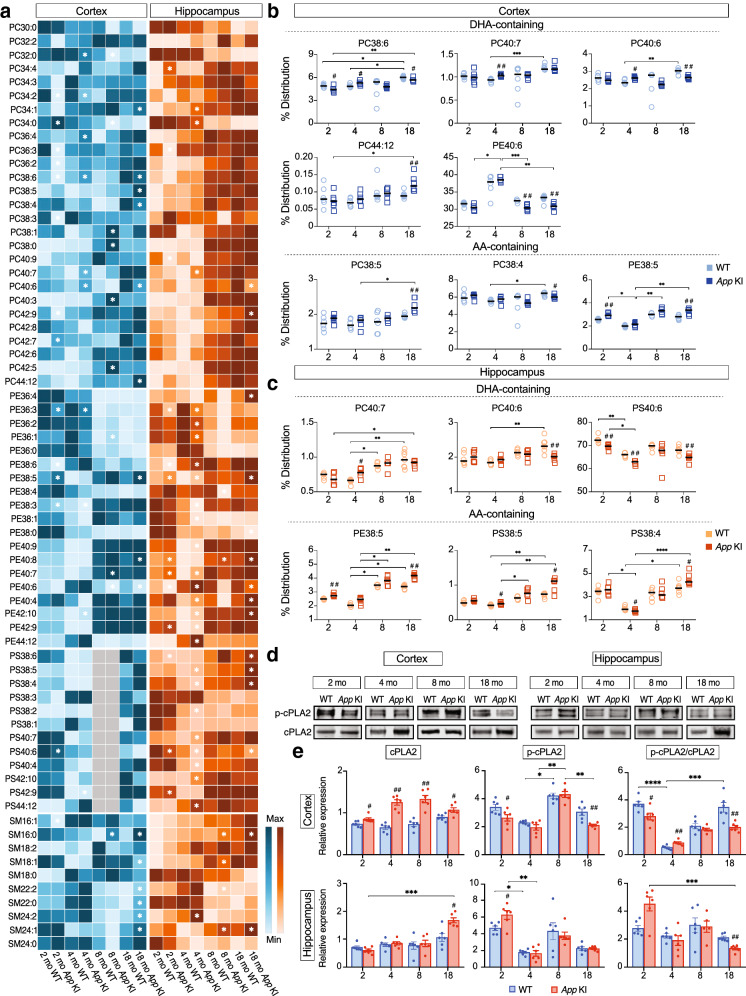
**Deficiency in DHA-containing phospholipids in the brain of App KI mice**. **a** Heat map analysis for PCs, PEs, PSs and SMs of 2, 4, 8 and 18 months-old WT (n = 4–6) and *App* KI mice (n = 6–7). Rows represent the mean values for phospholipids (PC, PE, PS, SM) and columns represent different ages of WT and *App* KI mice. Grey colour represents undetected values. **b**, **c** Scatter plots for DHA- and AA-containing phospholipids. Horizontal bars indicate median. *App* KI mice showed a marked increase in AA-containing phospholipids and decrease in DHA-containing phospholipids in both cerebral cortex and hippocampus at 18 months of age. Comparisons between genotypes were performed with Mann–Whitney U test, ^#^*P* < 0.05, ^##^*P* < 0.01, ^###^*P* < 0.001. Comparisons between different ages and genotypes were performed by Kruskal–Wallis one-way analysis of variance test with Dunn’s multiple comparisons post hoc test, *P < 0.05, **P < 0.01, ***P < 0.001, ****P < 0.0001. **d** Western blots for cPLA2 and p-cPLA2 in cortex and hippocampus. **e** Densitometric analysis of representative bands showed increasing levels of cPLA2 with age in *App* KI mice and a decrease in the phosphorylation rate of cPLA2. The data represent means ± SEM from 6 animals/group. Genotype comparisons were applied using Mann–Whitney U test (^#^*P* < 0.05, ^##^*P* < 0.01, ^###^*P* < 0.001) and multiple comparisons were performed with Kruskal–Wallis test and Dunn’s post hoc test (*P < 0.05, **P < 0.01, ***P < 0.001, ****P < 0.0001). PC = phosphatidylcholine, PE = phosphatidylethanolamine, PS = phosphatidylserine, SM = sphingomyelin, AA = arachidonic acid, DHA = docosahexaenoic acid, EPA = eicosapentaenoic acid, p-cPLA2 = phosphorylated (ser505) cytosolic phospholipase 2

We found alterations in fatty acyl chain composition of the phospholipids as a function of age in both *App* KI and WT mice, mainly at 18 months age. A most notable finding is that DHA-containing PCs (16:0/22:6 and 18:0/22:6) and PE (18:0/22:6) were lower in *App* KI compared to WT mice at 18 months in the cortex, whereas only PC (18:0/22:6) and PS (18:0/22:6) were decreased in the hippocampus (Fig. 4b, c). Remarkably, PC (44:12), which contains DHA for both sn-1 and sn-2, was higher in *App* KI compared to WT mice at 18 months (Fig. 4b). At earlier ages, PC (16:0/22:6) was lower in *App* KI at 2 months but higher at 4 months of age (Fig. 4b). The PCs (18:1/22:6 and 18:0/22:6) followed the same trend, showing increased levels in cortex at 4 months in *App* KI compared to WT mice, whereas in the hippocampus, only PC (18:1/22:6) showed a difference (Fig. 4b, c). In contrast, PS (18:0/22:6) levels were decreased at 2 and 4 months of age in the hippocampus of *App* KI mice (Fig. 4c).

Next, we observed that the AA-containing PC (18:1/20:4) and PE (18:1/20:4) were higher in the cortex of *App* KI compared to WT mice at 18 months, whereas PC (18:0/20:4) was lower in *App* KI mice (Fig. 4b). Similarly, PE (18:1/20:4), PS (18:1/20:4) and PS (18:0/20:4) were elevated in the hippocampus of *App* KI mice at 18 months of age, but there was no difference in AA-containing PCs (Fig. 4c). At 2 months, the AA-containing PE (18:1/20:4) was higher in both cortex and hippocampus of *App* KI mice (Fig. 4b, c). At 4 months, PS (18:1/20:4) was higher and PS (18:0/20:4) was lower in the hippocampus of *App* KI mice (Fig. 4c).

cPLA2 is one of the major PLAs in the brain that cleaves acyl chains from sn-2 of the glycerol backbone of phospholipids, exhibiting a preference for arachidonoyl acyl chains [14, 22]. We observed that the total cPLA2 levels were higher in the cortex of *App* KI mice at all ages. In the hippocampus, the cPLA2 levels were increased only at 18 months (Fig. 4d, e). We then evaluated total cPLA2 and phosphorylated cPLA2 (p-cPLA2) protein expression and found that the phosphorylated form of the enzyme was lower in the cortex of *App* KI mice at 2 and 18 months, while in the hippocampus p-cPLA2 levels were higher only at 2 months. Interestingly, the phosphorylation rate of cPLA2 (i.e. p-cPLA2/cPLA2 ratio) was decreased in both cortex and hippocampus at 18 months in the *App* KI mice. However, at 2 months, the p-cPLA2/cPLA2 levels were lower and at 4 months higher in the cortex of *App* KI mice (Fig. 4e).

cPLA2 is translocated to the perinuclear membrane upon binding of Ca^2+^ ions, followed by phosphorylation on serine residues by mitogen-activated protein kinases (MAPKs) [60]. This signaling is engaged in inflammation, cell death, and cell survival [86]. The phosphorylation rates of ERK1/2, p-38, and JNK were lower in the cortex of 18-month-old *App* KI compared to WT mice (Additional file 2: Fig. S2), which may explain the decrease in p-cPLA2 at 18 months. In the hippocampus, however, we found lower levels in *App* KI mice of p-ERK1/2 at 18 months, p-p38 at 4 months, and p-Akt at 8 months. The levels of p-JNK were decreased in the hippocampus of *App* KI mice at 8 months of age (Additional file 2: Fig. S2).

### Alterations in DHA- and AA- containing phospholipids within the hippocampus in relation to amyloid pathology

**Fig. 5:**
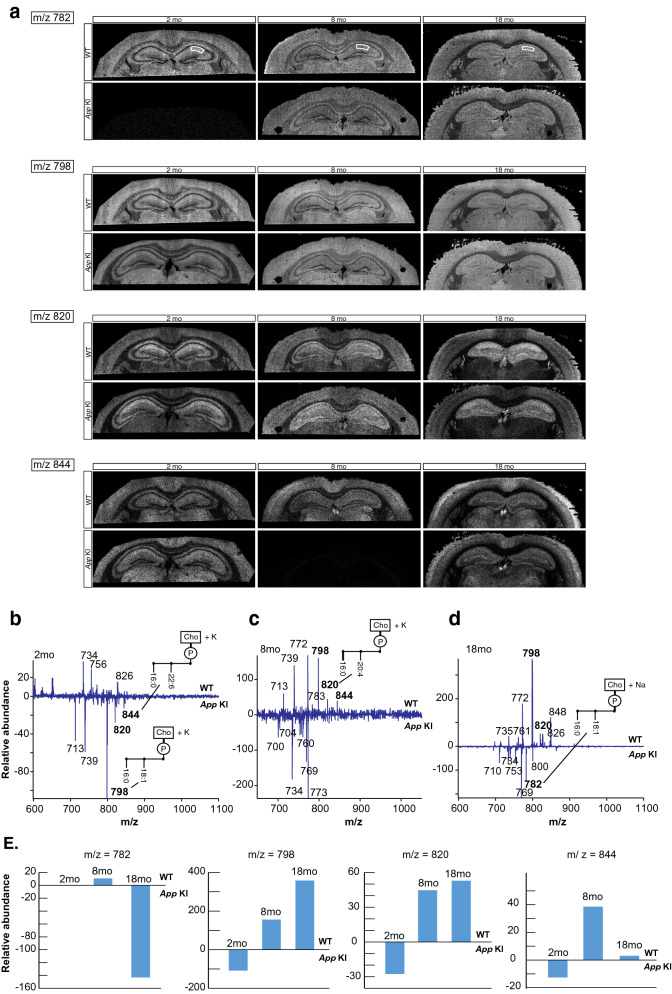
**Differential MALDI spectra reveal compensatory PCs generated in App KI hippocampus region**. **a** Lipid images of coronal mouse brain sections generated from positive ion mode data, illustrating the difference of abundance of specified lipids (m/z = 782, 798, 820 and 844) according to the age (2, 8 and 18 months) and phenotype of the mice (WT and *App* KI). The sections are of similar coronal levels of the brain. Encircled areas correspond to the region from which the lipid spectra were extracted, i.e. stratum radiatum within the CA1 region of each individual brain section to generate the differential MALDI spectra in B-D. **b**–**d** The differential spectra were constructed by subtracting the *App* KI profile from the WT profile and the resulting plots show relative abundances of the prevalent lipids in WT (pointing up) and lipids in *App* KI (pointing down). Molecules that are more abundant in *stratum radiatum* of WT mice are presented in the upper part of each graph, while molecules more abundant in *App* KI are displayed in the lower part. The different molecules show fluctuation of their relative abundance with age. **e** The difference of abundance of four molecules (m/z = 782, 798, 820 and 844) that display a switch in their level as the animal ages are shown, where m/z = 782 and 798 correspond to the same molecule PC (16:0/18:1) associated with Na^+^ adduct and K^+^ adduct, respectively, while m/z = 820 corresponds to the AA-containing PC, PC (16:0/20:4) associated with K^+^ adduct, and m/z = 844 corresponds to DHA-containing PC, PC (16:0/22:6) associated with K^+^ adduct. CA1 = *Cornu Ammonis 1*, MALDI-IMS = matrix-assisted laser desorption/ionization-imaging mass spectrometry, PC = phosphatidylcholine, WT = wild-type

To define differences in the lipidome of the WT and *App* KI mice within the hippocampus, brain sections of similar coronal level from the different ages of WT and *App* KI mice were imaged by MALDI-IMS. Although the images clearly show subregions of the hippocampus, due to the high spatial resolution (15 µm), the images show phospholipids from all cell types (neurons, glia, blood vessel endothelial cells) present in the analysed area. Total spectra from m/z 600 to m/z 1100 were collected from *stratum radiatum* within the CA1 region of the hippocampus (Fig. 5a) and a difference spectrum was constructed by subtracting the *App* KI profile from the WT profile (Fig. 5b–d). The resulting plots, while not quantitative, emphasized lipid abundance, with the prevalent lipids in WT (pointing up) and *App* KI (pointing down) mice.

Two lipids stand out particularly, PC (16:0/18:1) + Na^+^/ + K^+^ (m/z = 782 and 798, respectively) and PC (16:0/20:4) + K^+^ (m/z = 820) (Fig. 5e) and are therefore illustrative of the lipid changes in this region of the brain. Interestingly, the differential spectra of MALDI-IMS reveals that the *App* KI mice had a higher abundance of m/z = 798 in the earlier age compared to WT mice, and as the animals age, this lipid associated with K^+^ adduct accumulated more in the WT mice. In contrast, the same lipid associated with Na^+^ adduct (m/z = 782) accumulated more in the aging *App* KI animal as opposed to WT (Fig. 5e). A similar observation of lipid variation across the age and phenotype of the animal was made with m/z = 820; this AA-containing PC showed a higher accumulation with age in WT mice.

To evaluate DHA-containing PC, we have investigated the abundance of m/z = 844 PC (16:0/22:6) + K^+^ and found that at 2 months of age this lipid was more abundant in the *App* KI mice, but at 8 months it was detected in higher levels in the WT mice, while at the oldest age the level was only slightly higher in WT mice. Overall, this comparison suggests that the mechanism of the progression of the AD-pathology resides in molecular lipid variations early in the development, followed by an accumulation of these changes during aging, and that these variations could be further attributed to the ionic composition of the intracellular and extracellular environment of the neurons and glial cells.

### Marked increase in brain pro- and anti-inflammatory cytokines/chemokines at 18 months of age

**Fig. 6:**
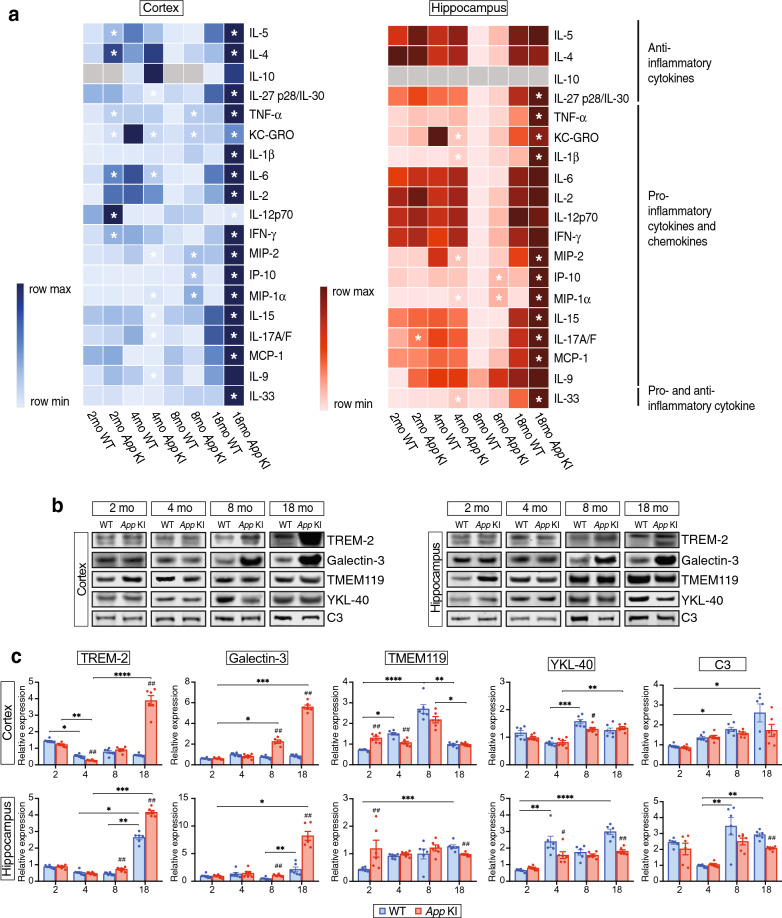
***App*** **KI mice show most marked changes in** **inflammatory factors at 18 months of age.** **a** Cytokines and chemokines were analyzed in homogenates of cerebral cortex and hippocampus by Meso scale v-plex assay. Rows in the heat map represent cytokines/chemokines and the columns represent different ages of WT and *App* KI mice. The colours represent mean of normalized concentration values (light blue and light red are low and dark blue and dark red are high). Grey colour represents undetected values. Sample size in each group was 5–6. Asterisks denote statistical significance between WT and *App* KI mice, where *P* < 0.05 using Mann–Whitney U test. **b** Western blot analysis of TREM-2, Gal-3, TMEM119, YKL-40 and C3 in cortex and hippocampus of 2-, 4-, 8- and 18-months-old mice (n = 6/group). **c** Densitometric analysis of bands after normalization with total protein and internal control. Intergroup comparisons were performed with Mann–Whitney U test (^#^*P* < 0.05, ^##^*P* < 0.01, ^###^*P* < 0.001). Multiple comparisons were performed with Kruskal–Wallis one-way analysis of variance test and Dunn’s post hoc test (*P < 0.05, **P < 0.01, ***P < 0.001, ****P < 0.0001). TREM-2 = triggering receptor expressed on myeloid cells 2, Gal-3 = galectin-3, TMEM119 = transmembrane protein 119, YKL-40 = chitinase-3-like protein 1, C3 = complement component 3

We next investigated whether cytokine and chemokine levels were altered in relation to age and pathology in a similar fashion as the bioactive lipids using multiplex analysis for 19 cytokines and chemokines (Fig. 6a). Pro-inflammatory chemokines (MIP-1α and MIP-2) and cytokines (IL-1β, TNF-α, IP-10) showed increases with age in both WT and *App* KI mice (Fig. 6a, Additional file 3: Fig. S3). Furthermore, most of the pro- and anti-inflammatory cytokines showed the highest levels in 18 months old *App* KI mice (Additional file 3: Fig. S3).

Heat maps of the data show that both pro- (IL-1β, TNF-α, IP-10, KC-GRO, IL-6, IL-2, IL-12p70, IFN-γ, MIP-2, MIP-1α, IL-15, IL-17A/F, MCP-1, and IL-33) and anti-inflammatory (IL-4, IL-5, and IL-27 p28/IL-30) factors were significantly increased in the cortex of 18-month-old *App* KI mice compared to WT mice (Fig. 6a). In the hippocampus, the pro-inflammatory factors (IL-1β, TNF-α, IP-10, KC-GRO, MIP-2, MIP-1α, IL-15, IL-17A/F, MCP-1, and IL-33) and IL-27 p28/IL-30, were significantly higher in 18-month-old *App* KI compared to WT mice (Fig. 6a). Surprisingly, the cytokine IL-33, shown to be decreased in human AD brains [12], was elevated in 18-month-old *App* KI mice, but a significant increase was also seen in 18 months old WT mice.

### Differential expression of microglial and astrocyte markers

We proceeded to investigate markers for an inflammatory phenotype expressed by microglia and astrocytes, i.e. triggering receptor expressed on myeloid cells-2 (TREM-2), galectin-3 (Gal-3), transmembrane protein 119 (TMEM119), chitinase-3-like protein 1 (YKL-40), and complement component 3 (C3). Apart from TM119, the microglial markers TREM-2, Gal-3 and C3 showed an increase with age in both cortex and hippocampus (Fig. 6b, c), and in the case of TREM-2 and Gal-3 this was seen only in the *App* KI mice, except for the hippocampus where there was also a marked increase in TREM-2 in 18 months old WT mice. At this age, the TREM-2 and Gal-3 levels were higher in *App* KI than in WT mice in both regions, and Gal-3 was also higher in the *App* KI mice at 8 months whereas TREM-2 only showed this difference at 8 months in the hippocampus. In the cortex, TREM-2 levels were lower in *App* KI mice compared to WT mice at 4 months, when both groups showed a decrease compared to 2 months.

The complement factor C3 showed a decrease in *App* KI mice compared to WT mice in the hippocampus at 18 months (Fig. 6c).

TMEM119, a marker for resident microglia [74], exhibited high levels in the cortex of both *App* KI and WT mice at 8 months age (Fig. 6c). An increase in *App* KI mice was observed in both cortex and hippocampus at 2 months, but a decrease was found in the cortex at 4 months and in the hippocampus at 18 months compared to WT mice.

YKL-40, expressed by astrocytes, unexpectedly displayed lower levels in *App* KI mice at 8 months in cortex and at 4 and 18 months in hippocampus (Fig. 6c).

### Differential activation of microglia and astrocytes upon increased amyloid pathology in *App* KI mice

**Fig. 7:**
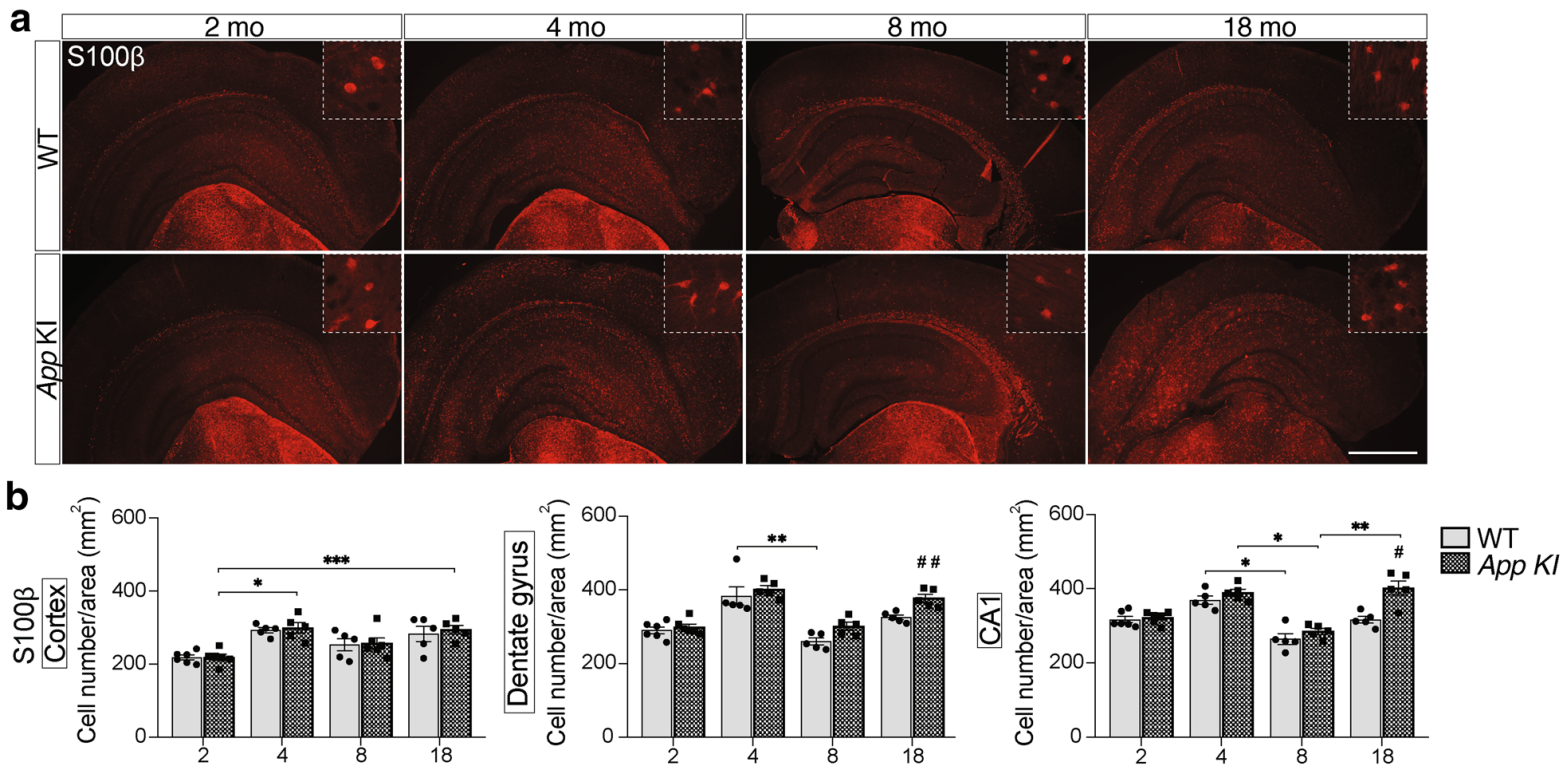
**Overall distribution of cortical astrocytes unchanged in App KI mice.** **a-b** The total number of S100β-positive cells in the cerebral cortex *App* KI mice was not different from that in WT mice, in contrast to the difference seen with GFAP (see Additional file 4: Fig. S4C-D) at 18 months age. In hippocampus (dentate gyrus and CA1), however, there was a significantly higher number of both S100β-positive and GFAP-positive (see Additional file 4: Fig. S4C-D) cells in the 18 months old *App* KI mice. S100β-positive cells were analyzed in 6 fields per section at 20 × magnification and two sections per animal. Cell numbers were normalized to area (mean ± SEM) (n = 5–6/group). Mann–Whitney U test was used for comparisons between genotypes, ^#^*P* < 0.05, ^##^*P* < 0.01, ^###^*P* < 0.001, and Kruskal–Wallis one-way analysis of variance test with Dunn’s post hoc test for comparisons between ages and genotypes, *P < 0.05, **P < 0.01, ***P < 0.001. Scale bars = 30 and 300 μm. S100β = S100 calcium-binding protein β, CA1 = Cornu Ammonis 1, GFAP = glial fibrillary acidic protein

Reactive gliosis is a well-established pathological process shown to be involved in AD pathogenesis [1, 5]. To determine the extent of the microgliosis and astrogliosis in relation to increasing AD-like pathology, sections from 2-, 4-, 8-, and 18-month-old WT and *App* KI mouse brains were processed for immunohistochemistry with the markers S100β (Fig. 7), Iba1 and GFAP (Additional file 4: Fig. S4). *App* KI mice exhibited significantly increased numbers of Iba1-positive microglia within the cerebral cortex and the CA1 at 4, 8, and 18 months compared to WT mice. In the dentate gyrus (DG), the microglia count was higher in *App* KI mice only at 18 months of age (Additional file 4: Fig. S4B). From 4 to 18 months of age, Iba1-positive microglia displayed a clearly activated phenotype with increased Iba1-immunoreactivity, retracted processes, and amoeboid appearance in clusters of cells (Additional file 4: Fig. S4A).

We then analyzed sections incubated with the astrocyte markers GFAP and S100β. There was a significant increase in GFAP-positive astrocytes in all regions analyzed (cerebral cortex, DG, CA1) of 18-month-old *App* KI mice compared to WT (Additional file 4: Fig. S4D). Surprisingly, younger ages did not show any difference between WT and *App* KI mice, but at 8 months of age, the number of GFAP-positive cells was higher in the cortex of *App* KI mice (*p* = 0.051) (Additional file 4: Fig. S4D). We also observed that there were fewer GFAP-positive cells in the cortex than in the DG and CA1 in WT mice (Additional file 4: Fig. S4C). The S100β-positive cells, however, were more evenly distributed (Fig. 7a), and analysis of cell counts showed similar results as for GFAP. Thus, S100β-positive cells were significantly more numerous in the DG and CA1 of *App* KI mice compared to WT mice at 18 months of age (Fig. 7b). However, there was no difference between WT and *App* KI mice with regard to the number of S100β-positive cells in the cortex (Fig. 7b).

### Effects of age on LMs and fatty acid acyl chain composition of phospholipids—relation to amyloid

Age-related changes in LMs and PUFA-containing phospholipids are visualized in heatmaps displaying the development of Aβ plaque load (Additional file 5: Fig. S5). Proportions of AA (38:5, 38:4)- and DHA (38:6, 40:7, 44:12)-containing PC, PE and PS were increased with age in both cortex and hippocampus of WT mice. In contrast, PS and PE 40:6 were at the highest levels at 2 and 4 months of age, respectively. A similar age-dependent pattern was observed in the hippocampus of *App* KI mice (Additional file 5: Fig. S5B). However, in the cortex of *App* KI mice, only PC 38:5, PC 40:7 and PC 44:14 showed a gradual increase. Other AA- and DHA-containing phospholipid classes demonstrated alterations at different time points (Additional file 5: Fig. S5A).

The bioactive LMs showed a similar age-dependent development in the hippocampus of WT and *App* KI mice, where most of the LMs (RvE1, MaR1, NPD1, LTB4, PGD2, PGF2a, 12-HETE, 15-HETE, 14-HDHA, 17HDHA, 20-HDHA) displayed a gradual increase from 2 months, the highest levels at 8 months, and then a drop at 18 months. In the cortex of the *App* KI mice, however, these LMs remained at high levels at 18 months (Additional file 5: Fig. S5).

### Importance of phospholipids and inflammatory proteins to discriminate between *App* KI and WT mice is dependent on age—multivariate discriminant model

**Fig. 8:**
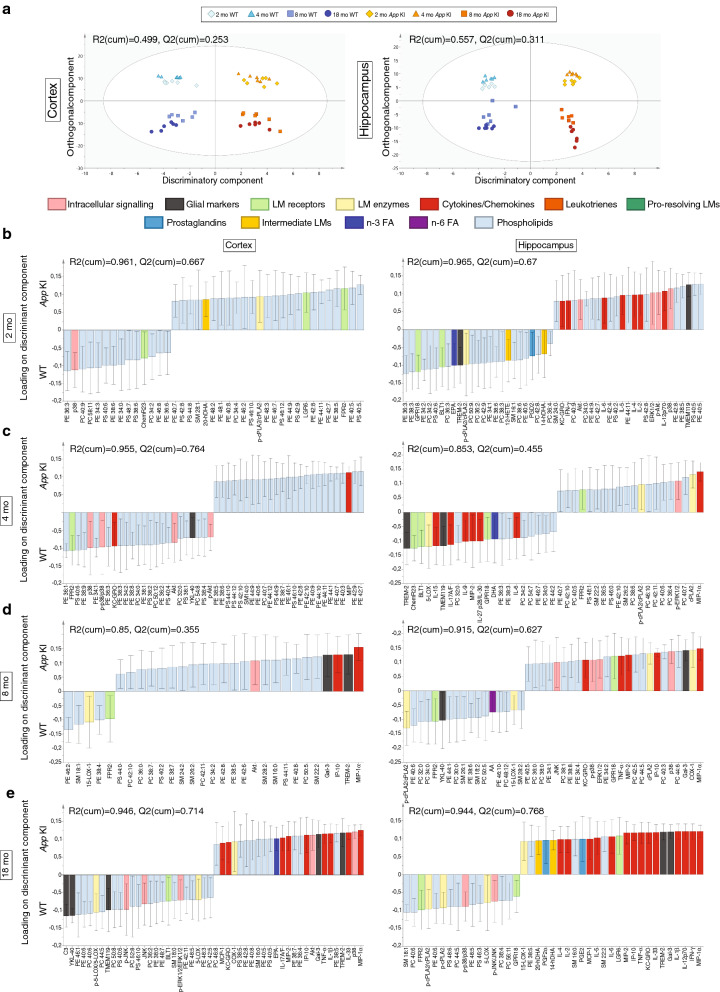
**Multivariate discriminant modelling of protein and lipid mediators, enzymes, receptors and phospholipids in the cortex and hippocampus from WT and App KI mice of different ages.** Orthogonal projections to latent structures (OPLS) – discriminant analysis (DA) was used to create models based on all ages (**a**), as well as of animals at 2 (**b**), 4 (**c**), 8 (**d**), and 18 (**E**) months of age, respectively. The quality of the models was determined by cross-validation, producing measures of the ability of the models to explain the variability of the data (R2(cum)) and to predict, i.e. discriminate between WT and *App* KI mice (Q2(cum)), as shown in the corresponding plots. The scatter plots in **a** show the distribution of animals of all ages along the discriminatory component (X-axis), and the first orthogonal component (Y-axis) for hippocampus and cortex, respectively. In **b–e**, the loadings on the discriminatory component are shown from the OPLS-DA models based on data from each age and separated according to region. Only factors with a significant impact (with a standard error of the loadings from the cross-validations that does not shift sign, i.e. does not cross the X-axis) are shown and limited to the 25 factors with highest positive and 25 with highest negative loading (higher levels favouring categorization as *App* KI and WT, respectively), except for models in which 25 factors with significant loading could not be found

To further investigate how the levels of lipid and protein mediators, enzymes, receptors, and phospholipids differ between WT and *App* KI mice at different ages, a multivariate approach was employed (Fig. 8), providing a means of analyzing the relationship between many factors simultaneously by estimating how well their covariance can be mathematically explained by introducing a new dimension(s) upon which the variables are projected. We used orthogonal projections to latent structures (OPLS)—discriminant analysis (DA) to produce a model to elucidate which factors are important for discriminating between WT and *App* KI mice, as well as how the covariance can be explained by a component orthogonal to the discriminatory component, i.e. variability in the data not related to genotype.

First, separate OPLS-DA models of cortex and hippocampus including animals of all ages were produced (Fig. 8a). For both regions, there was a distinct separation of animals according to genotype on the discriminant (X) component. Cross-validation showed that the models were of reasonably good quality (R2(cum) = 0.499, Q2(cum) = 0.523 for cortex, and R2(cum) = 0.557, Q2(cum) = 0.311 for the hippocampus). However, the case scatter plots of the animals in Fig. 8a clearly show a separation along the orthogonal (Y) component according to age, supporting the results from univariate statistics showing that the age of the animals is an important determinant for the pattern of levels of proteins, lipids, and phospholipids. Therefore, to elucidate the discriminative influence of the factors on genotype more distinctly we produced age-specific OPLS-DA models.

The eight resulting models were of very good quality as shown by cross-validation, with R2(cum)values close to 1, and Q2(cum)-values around 0.7. The models seen in Fig. 8b, c suggest an age-related shift, where phospholipids are the most important class of factors for discrimination in the youngest animals (Fig. 8b) and inflammatory protein and peptide factors are most important in the oldest (18-month-old) animals (Fig. 8c). For example, in 2-month-old animals, the presence of high levels of PS (40:5) in the cortex and hippocampus was highly influential in discriminating (classifying) animals as *App* KI, while PE (36:3) and PS (40:6) drove classification as WT. In contrast, high levels of the inflammatory proteins MIP-1α, IL-1β and Gal-3 were important factors in both cortex and hippocampus for classification as *App* KI in 18-month-old animals.

The pattern influencing classification as WT was less clear and less consistent between the regions. In the cortex, high levels of the astrocytic markers C3 and YKL-40 drove the classification as WT, while in the hippocampus, a mix of phospholipids (SM (18:1) and PC (40:6)), LM receptors (FPR2 and GPR18), enzymes involved in lipid synthesis (cPLA2 and 5-LOX), and activation of inflammatory stress pathway proteins (p-p38/p38 and p-JNK/JNK) drove the classification as WT.

Hence, the MVA OPLS-DA model strongly support the results from the univariate statistical analysis, highlighting the dominant influence of aging on the levels of factors, while also showing that factors important for discriminating between WT and *App* KI mice to a large extent overlap with factors that showed significant difference in univariate comparison.

## Discussion

The void in our current understanding of the factors that modulate neuronal survival signaling in the aging brain as well as during AD pathogenesis was tackled here in several ways. We defined phospholipid composition of cell membranes that form a proper milieu for the function of key proteins as well as being storage sites of LM precursors during aging and in an AD model. In addition, we found that pro-inflammatory and pro-resolving LMs do not change until advanced age in the WT mice and in the AD model, despite Aβ production. Furthermore, the LM biosynthetic enzymes were increased, and their receptors decreased in the oldest *App* KI mice. Because AA-containing phospholipid molecular species were elevated in the AD model, and spatial molecular imaging of phospholipids depicted differential distribution according to genotype in the hippocampal layers we suggest that this reflects decreased cPLA2 activity. Histology unveiled the early onset of microglia proliferation in the *App* KI mice, while astrocyte numbers were augmented in older ages. Interestingly, the proliferation and morphological signs of microglial activation were not accompanied by increases in inflammatory mediators until the oldest age. The multivariate analysis clearly demonstrated an age-related shift in the pattern of differences between WT and *App* KI, with increased inflammation in the cortex and hippocampus of *App* KI mice with age, while differences in phospholipid content became less prominent. Furthermore, the multivariate analysis showed that loadings on the discriminatory component corresponded with univariate analysis in many cases (Gal-3, TREM-2, C3, and PC (18:0/22:6)).

Age-related changes in brain lipids have given inconsistent results in human and animal studies [17, 96]. One of the main reasons for this has been that, unlike the present study, individual phospholipids and their molecular species were often not studied. In cortex and hippocampus of WT mice we found a gradual increase in AA- and DHA-containing PC, PE, and PS classes with age, indicating that phospholipid composition follows a similar pattern between two regions [23]. In the *App* KI mice, the aging process had a different effect in the cortex and hippocampus on the proportions of PUFAs in phospholipid classes. This indicates that the alterations in PUFA composition of the AD brain are due to a slower or inadequate aging than in healthy brains [89]. In the present study, hippocampus of the *App* KI mice showed a gradual elevation for AA- and DHA-containing phospholipids, where 18-month-old mice had the highest levels compared to younger ages. However, in the cortex of *App* KI mice, this pattern is not followed, which may indicate that the hippocampus of *App* KI mice is influenced by Aβ pathology and may have a higher resistance to oxidative stress than the cortex and is more similar to the WT mice [80, 95].

There are age-related changes in the biosynthesis of LMs for both WT and *App* KI mice with the largest alterations in LM abundance at 8 months of age for both groups of mice, and a gradual increase starting at 2 months. Intriguingly, the LMs drop at 18 months of age. However, the cortex of the aged *App* KI mice showed high levels of intermediate LMs, as well as some of the pro-resolving LMs indicating that the pathways are turned-on, however not yet fully active. This supports our hypothesis that the hippocampus may have a more effective oxidative defense, indicating the vulnerability of the cortex [83]. Thus, in the *App* KI mice, the cortex and hippocampus are not equally affected during aging with pathology. Due to the advanced pathology, the cortex may be attempting to provide neuronal survival with the synthesis of LMs at old age, more than the hippocampus.

Pro-inflammatory LMs followed by pro-resolving LM production at the peak of inflammation counteract inflammatory damage [61, 78]. Deficiency in inflammation resolution fosters chronic inflammatory diseases, including AD and MS [18, 38, 49, 99], but alterations in LMs have not been studied from a perspective of aging and AD disease progression. Therefore, we have correlated our data with aging and the progression of AD pathology by defining levels of LMs (intermediate, pro-inflammatory and pro-resolving) and their biosynthetic enzymes and multi-ligand receptors in WT and *App* KI mice at different ages. We found that pro-inflammatory (PGD_2_, PGE_2_, PGF_2α_, and LTB4) as well as pro-resolving (RvE1, NPD1, and LXA_4_) LMs were higher in *App* KI mice than in WT mice at 18 months. PGE_2_, LTB4, and LXA_4_, derived from AA, are initiators of ‘class-switching’ of the biosynthetic production from AA to DHA and EPA [44], and their increase implies that inflammation resolution begins at 18 months in the *App* KI mice as an attempt to slow down damaging inflammation, as evidenced by the production of pro-resolving LMs (RvE1 and NPD1). Our observations on biosynthetic enzymes and receptors for LMs further support this concept. COX-1, involved in prostaglandin (PG) production [64], increases with age [57], as confirmed by our results for both WT and *App* KI mice. Furthermore, the levels of COX-1 in cerebral cortex and hippocampus were higher in 18-month-old *App* KI mice than in WT mice, correlating with the elevated levels of PGs. AD brains showed increased expression of 15-LOX-1 [104], and similarly, we found higher levels of this enzyme in the *App* KI mice at an age (18 months) when an increase in 15-HETE, 17-HDHA, and LXA_4_ levels, all of which are products of 15-LOX-1 enzyme activity, takes place [8, 29, 52]. Upon translocation to the nuclear envelope, 5-LOX mediates the production of pro-inflammatory LMs, but when phosphorylated at Ser523, this translocation is prevented [50], leading to decreased leukotriene production and a class-switch towards the production of pro-resolving LMs. Furthermore, our findings of increased LTB_4_ levels in the *App* KI mice at 18 months and decreased p-5-LOX levels in cortex and hippocampus are consistent with a pro-inflammatory phenotype in the brain of older *App* KI mice.

The differential expression of bioactive LM receptors discloses that FPR2, mediating the activities of LXA_4_ and RvD1, was decreased in the cortex of *App* KI mice at 8 and 18 months, suggesting a negative feedback response to the elevated LXA_4_ levels. Interestingly, Aβ is a ligand for this receptor [93], and the increased levels of Aβ may contribute to the negative feedback. Earlier studies have demonstrated a reduction in FPR2 levels in an AD mouse model and an in vitro model [66]. BLT1 and ChemR23, receptors for RvE1, were also found to be reduced in the *App* KI mice, and the increased levels of RvE1 suggests a compensatory mechanism [20, 99]. It is noteworthy that our previous studies in human *post mortem* brains showed increased levels of these receptors in AD [20, 99]. However, the stage of AD pathology in 18-month-old *App* KI mice is conceivably less advanced compared to that seen in human *post mortem* brains, which may explain the discrepancy. Together with previous studies, our data using the *App*^*NL−G−F*^ knock-in mouse model suggest that at early stages in AD, resolution is initiated by increasing pro-resolving LM production and LM receptor expression. Due to Aβ accumulation and the ensuing chronic inflammation, resolution fails, pro-resolving LM levels drop, and the expression of their receptors increases as a response to under-stimulation. In contrast, the levels of LGR6, a receptor for MaR1 [13], were higher in *App* KI than in WT mice in all ages studied. LGR6 is expressed in phagocytes, and MaR1 induces phosphorylation of specific kinases in an LGR6-dependent manner [13]. Higher levels of LGR6 may reflect a compensatory mechanism to promote resolution in response to insufficient MaR1 availability.

To define the availability of precursors for the production of bioactive pro-resolving LMs in the brain, we analyzed phospholipids containing DHA and AA in the *App* KI mice, and showed that DHA-containing PCs, PEs, and PSs were reduced in the cortex and hippocampus of *App* KI mice, especially at 18 months, in agreement with data on human AD brain and other AD models [16, 65]. Conversely, AA-containing PCs, PEs and PSs were increased, also in line with previous studies [63]. These results indicate an imbalance between DHA and AA availability at 18 months in the *App* KI model. Therefore, decreased DHA and increased AA levels may contribute to the increased production of inflammatory LMs and the reduced formation of pro-resolving LMs in advanced ages, thus driving chronic inflammation.

To validate the changes in DHA- and AA-containing phospholipids, we analyzed the levels of cPLA2, an enzyme that preferentially cleaves AA from brain phospholipids [39]. Phosphorylation of cPLA2 at Ser505 by MAPK kinases enhances cPLA2 activity and the release of AA from phospholipids. Our analysis showed a decreased phosphorylation rate of cPLA2, consistent with the increased brain AA incorporation in *App* KI mice. Moreover, we observed increased levels of cPLA2 at all ages of the *App* KI mice, similar to observations in *post mortem* AD brains [40, 82]. cPLA2 is phosphorylated at Ser505 by either ERK1/2, p38, or JNK, the three main effectors of the MAPK cascade pathway, resulting in the increased specific activity of the enzyme [10, 19, 47]. The observed decrease in the phosphorylation rate of ERK1/2, p38, and JNK in *App* KI mice is in agreement with the reduced levels of p-cPLA2 and may reflect reduced levels of activation of the MAPK cascades or increased activity of phosphatases such as PP2A, able to dephosphorylate MAPKs or their upstream MAPKs [79, 87, 105].

Lipid profiling of tissue homogenates encompasses alterations within the entire cortex and hippocampus regions, and changes observed in phospholipids and LMs reflect neurons, glia and other cells. Moreover, lipid analysis using whole-brain tissue may mask cell-specific differences in lipid alterations in different regions of the CNS [90]. MALDI imaging investigates the localization of the changes in phospholipids within the brain, and with the current level of resolution of the method (15 µm) the MALDI-IMS allowed us to observe regional differences in molecular species of phospholipids in the hippocampal CA1 region of WT and *App* KI animals. It would be of great interest to decipher changes in lipid composition in different cell types. However, the images obtained by this technique show phospholipids from all cell types present in the analysed area and the exact cellular localization and profile of phospholipids remains to be determined by further improvements of the technique, and other approaches [43]. The results obtained showed that AA-containing PC, PC (16:0/20:4) + K^+^ accumulated with age in the WT mice, while the DHA-containing PC, PC (16:0/22:6) + K^+^ was widespread in the WT as the animal aged. MALDI-IMS data are not quantifiable and thus we only report the relative abundance of the most represented molecules. MALDI-IMS also highlighted variations in Na^+^/K^+^ relative abundance in the hippocampus of the animals. Previous MALDI studies have demonstrated a large abundance of protonated lipid species in healthy tissue, while an increase in lipids associated with Na^+^ and K^+^ adducts is correlated with the pathophysiology, where inflammation, gliosis, necrosis and apoptosis occur [24]. These changes in intracellular and extracellular Na^+^/K^+^ balance could be attributed to the activation of microglial voltage-gated sodium channels [6], lipid peroxidation by modifying the function of Na^+^/K^+^ ATPase [58], or could be linked to the channel hypothesis proposing that Aβ peptides form ion channels permissive for Ca^2+^, Na^+^, K^+^, Cs^+^, Li^+^, and even Cl^−^ entry [32].

Surprisingly, only a limited range of pro-inflammatory cytokines and chemokines was upregulated at an early age of the *App* KI mice, despite the production of Aβ already at 2 months. However, the early response pro-inflammatory cytokines [4, 59], TNF-α and IL-6, were increased at 2 months of age in the *App* KI mice, in agreement with early microglial activation, suggesting a response to Aβ peptide accumulation. Interestingly, anti-inflammatory cytokines (IL-4, IL-5, and IL-27 p28/IL-30) were also upregulated at 2 and 4 months of age indicating an attempt to resolve inflammation at an early stage of Aβ pathology. IL-4 suppresses TNF-α and IL-1β production [28], while IL-5 promotes survival and growth [91] and IL-27 p28/IL-30 induces production of the anti-inflammatory cytokine IL-10 [84]. Remarkably, the most prominent changes in cytokines/chemokines occurred at 18 months in the *App* KI mice, and the levels of these factors were markedly higher at 18 months than in younger ages.

TREM-2 is a transmembrane receptor crucial for regulating microglial inflammatory responses and phagocytosis [36], and mutations of TREM-2 are associated with high-risk AD development [35]. In line with previous reports [75], we showed upregulated TREM-2 levels in 8- and 18-month-old *App* KI mice, in support of a peak of inflammation at 18 months of age when microglia respond to advanced Aβ pathology in an attempt to increase phagocytic function. Additionally, increased levels of TREM-2 correlate with microglial proliferation, as indicated by decreased numbers of microglia after injury in TREM-2 and DAP12 (ligand for TREM-2) knock-out mouse models [37, 68].

Gal-3 is a newly identified ligand of TREM-2 [7], and our studies showed increased levels of Gal-3 in both the cortex and hippocampus of the *App* KI mice, in agreement with upregulation in mouse and human *post mortem* AD brains [92, 100]. TMEM119 is a marker for resident microglia [74], and our data showed an upregulation in both cortex and hippocampus in 2 months old *App* KI mice. This may indicate an increased number of resident microglia in response to the newly formed Aβ in the young *App* KI mice. The finding that TMEM119 levels were decreased in the older mice suggests that the microglia reach a phase of activation near phagocytic function, supporting previous studies [97].

Markers for astrocytes indicated a different pattern of activation. YKL-40, a glycoprotein expressed by astrocytes [70], is elevated in the brain and CSF of AD patients [20, 48] and is a potential diagnostic biomarker for neurodegenerative diseases [2, 15]. Increased expression of YKL-40 has been observed in cells with high cellular activity, and a role in tissue remodeling during inflammation has been suggested [42, 71, 72]. Unexpectedly, the YKL-40 levels were reduced in the *App* KI mice at 4, 8, and 18 months. A possible explanation may be that secretion of YKL-40 to the CSF and blood resulted in reduced levels in the brain. The reports of increased levels of YKL-40 in CSF and plasma were based on samples from early AD [15], and our results strongly indicate that the ages analyzed (2, 4, 8, and 18 months) represent the beginning of inflammation in the *App* KI. Studies showing increased levels of YKL-40 in *post mortem* AD brains are conceivably based on cases with advanced stages of AD.

C3 is an important complement factor for the crosstalk between microglia and astrocytes [46] and was found to be upregulated in AD [103]. Aβ-induced activation of nuclear factor κB (NF-κB) in astrocytes resulted in the release of C3, which acts on the C3a receptor in microglia [45]. Our studies showed decreased levels of C3 in the hippocampus of 18-months-old *App* KI mice. Complement activation leads to the production of functional fragments, with sequential cleavages from the conversion of C3, involved in phagocytosis via C3b opsonization and chemotaxis via C3a and C5a [98]. Thus, the decrease in C3 levels may be due to cleavage into fragments since the antibody used was against intact component C3 (185 kDa) but not the fragments, suggesting complement activation at 18 months in the *App* KI mice [94].

The *App* KI model presents microgliosis and astrogliosis that increase with age in the cortex and hippocampus [53]. Our data showed a higher cell count for Iba1-positive cells in the *App* KI mice, starting from 4 months and showing the most significant difference from WT mice at 8 and 18 months of age. Iba1 is widely used to show all forms of microglia, and there is ample evidence for an upregulation of Iba1 immunoreactivity during inflammation [62]. We observed abundant Iba1-positive microglia in the cortex and CA1, and clusters of microglia are seen in the vicinity of Aβ plaques [110] at 4, 8 and 18 months. The increase in the number of Iba1-positive microglia with age in *App* KI mice supports our finding of elevated TREM-2, a protein known to be involved in microglial proliferation [108].

Astrocytes undergo functional and morphological changes during aging, and recent reports address this heterogeneity within different brain regions [34]. Isolation of cells from specific brain regions for proteomics [11] and RNA profiling [9] revealed differences in gene expression and morphological features of astrocytes within individual brain regions [41]. GFAP is a marker for astrocyte activation [26] and cell count. However, histological analysis indicated that S100β as a cytosolic marker is more suitable for the analysis of overall distribution and cell count within the entire brain, while GFAP is mainly found to be expressed by astrocytes within the hippocampus [106]. Therefore, our analysis of astrocytes was performed using both of these markers, showing the estimated number of astrocytes labelled with S100β and the number of reactive astrocytes labelled with GFAP. Both markers showed an increase in astrocyte count in the hippocampus of 18-months-old *App* KI mice, in line with findings for C3. However, in the cortex, there was no difference in S100β-positive astrocytes between *App* KI and WT mice, whereas GFAP-labelling showed an increase in 18 months old *App* KI mice. These findings indicate that the advanced Aβ plaque-pathology in the cortex results in upregulation of GFAP expression by astrocytes due to their activation.

In conclusion, we uncover lipidomic profiles, including biosynthetic pathways and inflammatory factors in relation to increasing age and pathology of the *App* KI model in comparison with age-matched WT mice. Importantly, our data showed that both pro-inflammatory and pro-resolving LMs were elevated in 18 months old *App* KI mice, indicating a peak of inflammation and a class-switching mechanism. This also indicates that the production of Aβ at earlier ages did not induce a potent inflammatory response until the Aβ plaque pathology was excessive since the most pronounced alterations were observed at 18 months of age. Furthermore, the highest levels of cytokines and chemokines were observed in 18 months old *App* KI mice, similar to the findings on LMs and emphasizing the necessity of further investigation in even more advanced ages to see if pro-resolving LMs drop and inflammatory LMs continue to increase, as hypothesized based on human *post mortem* brain studies. The findings of this study open avenues to explore aging and disease mechanisms and the potential use of pro-resolving LMs to blunt inflammation onset at an early disease stage before inflammation becomes chronic.

## Supplementary Information

**Additional file 1: Fig. S1**. Analysis of LXA4 and MaR1 in cerebral cortex and hippocampus of App KI and WT mice. (A) Lipoxin A4 (LXA4) and maresin 1 (MaR1) were analyzed in the cerebral cortex and hippocampus of 2, 4, 8 and 18 months-old WT (n = 4-6) and App KI mice (n = 6-7) using LC-MS/MS. Horizontal bars indicate median. Groups below the limit of detection were not shown. Kruskal-Wallis with Dunn’s post hoc test was used for multiple comparisons (*P < 0.05, **P < 0.01).

**Additional file 2: Fig. S2**. Western blot analysis of ERK1/2, p38, Akt and JNK phosphorylation in cortex and hippocampus of WT and App KI mice at 2, 4, 8 and 18 months-age. Densitometric quantification of bands after normalization with total protein and internal control are shown. Data are expressed as mean ± SEM, 6 mice in each group, and statistical significance analysed by Mann-Whitney U test (#P < 0.05, ##P < 0.01, ###P < 0.001) and Kruskal-Wallis one-way analysis of variance test with Dunn’s multiple comparison post hoc test (*P < 0.05, **P < 0.01, ***P < 0.001). ERK1/2 = extracellular signal regulated protein kinases 1 and 2, Akt = protein kinase B, JNK = c-Jun N-terminal kinase.

**Additional file 3: Fig. S3**. Age comparison of cytokines and chemokines in 2, 4, 8 and 18 months-old mice for WT and App KI in the brain. Cytokines and chemokines were analyzed in homogenates of cerebral cortex and hippocampus by Meso scale v-plex assay. Data are expressed as mean ± SEM, 5-6 mice in each group and statistical analysis was performed with Kruskal-Wallis one-way analysis of variance test with Dunn’s multiple comparisons post hoc test, *P < 0.05, **P < 0.01, ***P < 0.001, ****P < 0.001.

**Additional file 4: Fig. S4**. (A, B) Sections stained for Iba1 show significantly higher number of Iba1-positive cells in App KI mice compared to WT mice starting at 4 months of age in cortex, DG and CA1. Cells were counted in three fields per section and two sections for each animal at 10x magnification. (C, D) Sections stained for GFAP show increased numbers of GFAP-positive cells in the cerebral cortex, DG and CA1 of 18 months old App KI mice compared to WT mice. Cells were counted in six fields per section and two sections per animal at 20x magnification. Cell numbers were normalized to area (mean ± SEM) (n = 5-6/group). Kruskal-Wallis one-way analysis of variance test with Dunn’s multiple comparison post hoc test *P < 0.05, **P < 0.01, ***P < 0.001; Mann-Whitney U test #P < 0.05, ##P < 0.01, ###P < 0.001). Scale bars = 30 and 300 μm. Iba1 = ionized calcium-binding adapter molecule 1, GFAP = glial fibrillary acidic protein, DG = dentate gyrus, CA1 = Cornu Ammonis 1.

**Additional file 5: Fig. S5**. Heat map analysis of bioactive LMs, AA- and DHA-containing phospholipids and amyloid levels in 2, 4, 8 and 18 months-old WT (n = 4-6) and App KI mice (n = 6-7). Rows represent the median values and columns represent different ages of WT and App KI mice. Grey colour represents undetected value.

## Data Availability

The datasets used and/or analyzed during the current study are available from the corresponding author upon reasonable request.

## Abbreviations

AA: Arachidonic acid
AD: Alzheimer’s disease
APP: Amyloid precursor protein
Aβ: β-Amyloid
BLT1: LTB_4_ receptor
CA1: *Cornu Ammonis 1*
ChemR23: Chemokine-like receptor 1
COX-1: Cyclooxygenase-1
CSF: Cerebrospinal fluid
C3: Complement component 3
DA: Discriminant analysis
DG: Dentate gyrus
DHA: Docosahexaenoic acid
EPA: Eicosapentaenoic acid
FPR2: Formyl peptide receptor 2
Gal-3: Galectin-3
GPR18: G-protein-coupled receptor 18
LC–MS/MS: Liquid chromatography-tandem mass spectrometry
LGR6: Leucine-rich repeat containing G protein-coupled receptor 6
LM: Lipid mediator
LTB_4_: Leukotriene B4
LXA_4_: Lipoxin A4
MALDI-IMS: Matrix-assisted laser desorption/ionization-imaging mass spectrometry
MAPK: Mitogen-activated protein kinase
MaR1: Maresin 1
MVA: Multivariate analysis
NPD1: Neuroprotectin D1
OPLS: Orthogonal projections to latent structures
PBS: Phosphate‐buffered saline
PC: Phosphatidylcholine
PE: Phosphatidylethanolamine
PF: Paraformaldehyde
PGD2: Prostaglandin D2
PGE2: Prostaglandin E2
PGF2a: Prostaglandin F2a
PS: Phosphatidylserine
RIPA: Radio immunoprecipitation assay
RT: Room temperature
RvE1: Resolvin E1
SM: Sphingomyelin
TMEM119: Transmembrane protein 119
TREM-2: Triggering receptor expressed on myeloid cells-2
WT: Wild-type
YKL-40: Chitinase-3-like protein 1
14HDHA: 14-Hydroxydocosahexaenoic acid
15-HETE: 15-Hydroxyeicosatetraenoic acid
15-LOX-1: 15-Lipoxygenase-1
