# Supplementary figures and images for: Age-related changes in brain phospholipids and bioactive lipids in the APP knock-in mouse model of Alzheimer’s disease
Source: Acta Neuropathol Commun. 2021 Jun 29;9:116. doi: 10.1186/s40478-021-01216-4 (PMC8244172; doi:10.1186/s40478-021-01216-4)

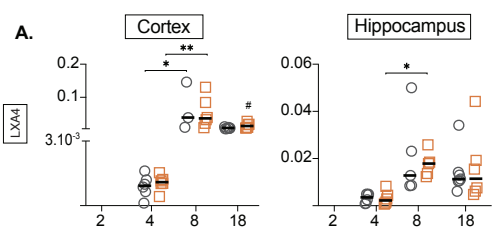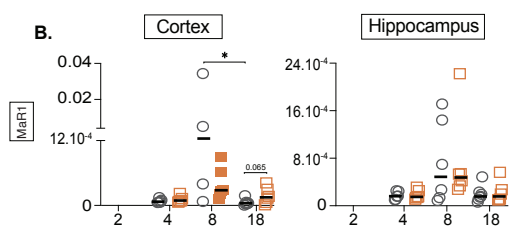

Supplement: Supplementary file 1 — Additional file 1: Fig. S1. Analysis of LXA4 and MaR1 in cerebral cortex and hippocampus of App KI and WT mice. (A) Lipoxin A4 (LXA4) and maresin 1 (MaR1) were analyzed in the cerebral cortex and hippocampus of 2, 4, 8 and 18 months-old WT (n = 4-6) and App KI mice (n = 6-7) using LC-MS/MS. Horizontal bars indicate median. Groups below the limit of detection were not shown. Kruskal-Wallis with Dunn’s post hoc test was used for multiple comparisons (*P < 0.05, **P < 0.01). [file 40478_2021_1216_MOESM1_ESM.pdf]

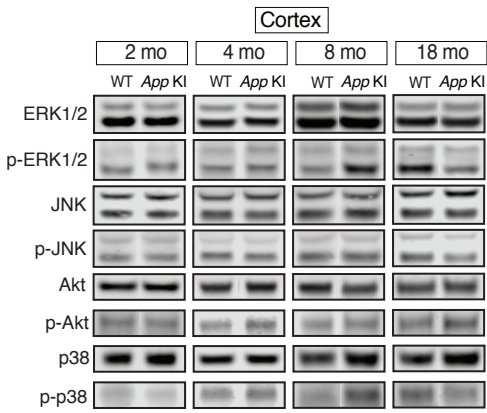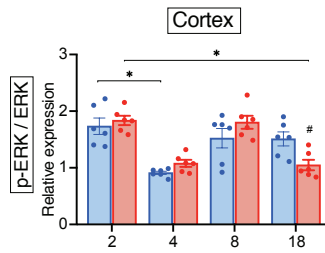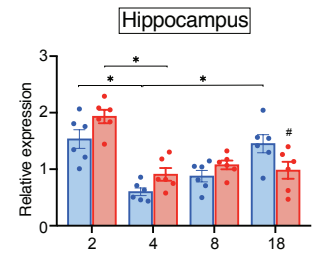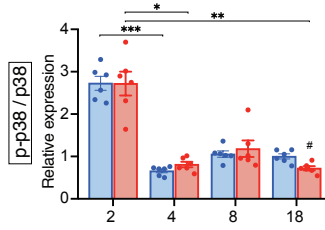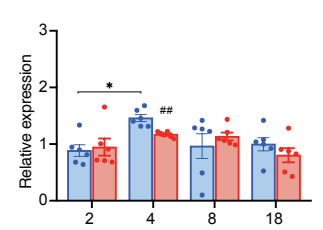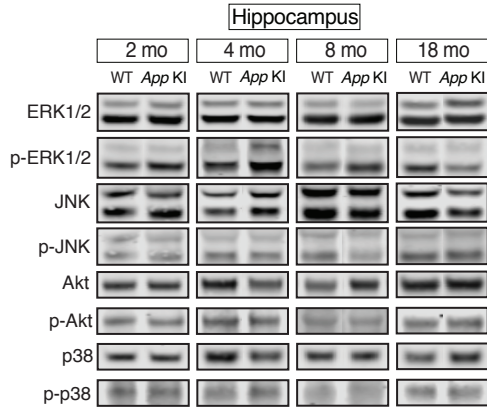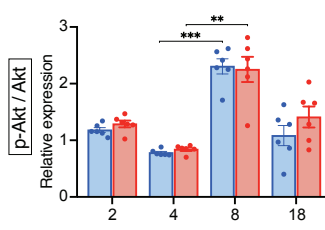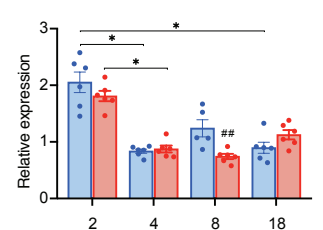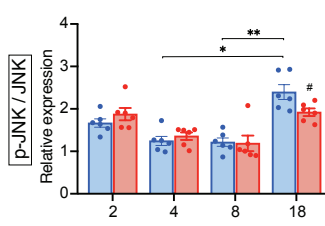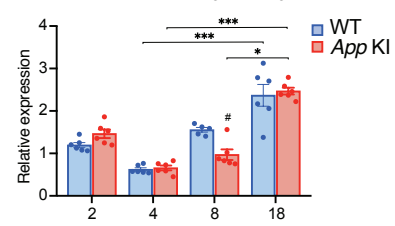

■ WT  
■ *App* KI

Supplement: Supplementary file 2 — Additional file 2: Fig. S2. Western blot analysis of ERK1/2, p38, Akt and JNK phosphorylation in cortex and hippocampus of WT and App KI mice at 2, 4, 8 and 18 months-age. Densitometric quantification of bands after normalization with total protein and internal control are shown. Data are expressed as mean ± SEM, 6 mice in each group, and statistical significance analysed by Mann-Whitney U test (#P < 0.05, ##P < 0.01, ###P < 0.001) and Kruskal-Wallis one-way analysis of variance test with Dunn’s multiple comparison post hoc test (*P < 0.05, **P < 0.01, ***P < 0.001). ERK1/2 = extracellular signal regulated protein kinases 1 and 2, Akt = protein kinase B, JNK = c-Jun N-terminal kinase. [file 40478_2021_1216_MOESM2_ESM.pdf]

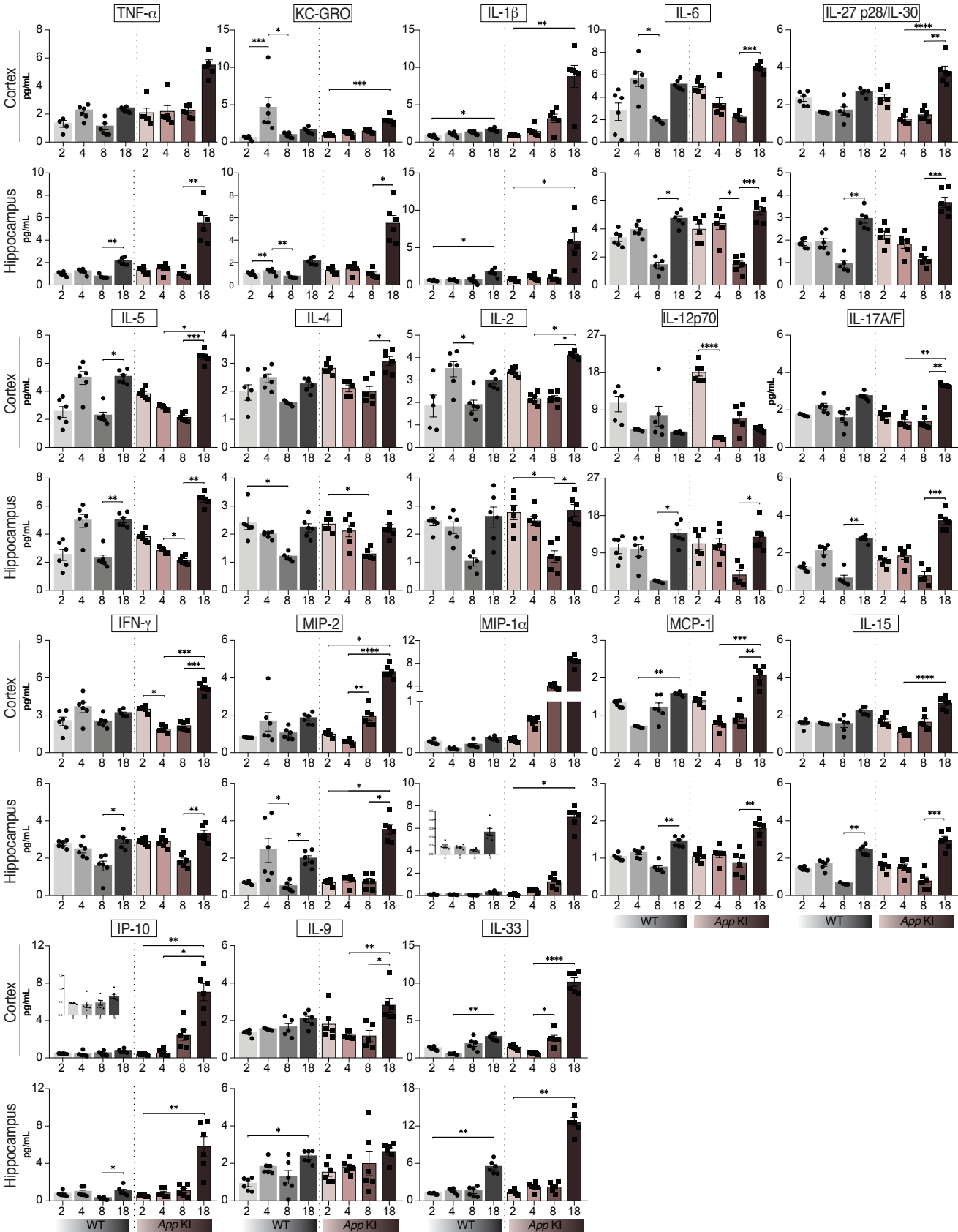

Supplement: Supplementary file 3 — Additional file 3: Fig. S3. Age comparison of cytokines and chemokines in 2, 4, 8 and 18 months-old mice for WT and App KI in the brain. Cytokines and chemokines were analyzed in homogenates of cerebral cortex and hippocampus by Meso scale v-plex assay. Data are expressed as mean ± SEM, 5-6 mice in each group and statistical analysis was performed with Kruskal-Wallis one-way analysis of variance test with Dunn’s multiple comparisons post hoc test, *P < 0.05, **P < 0.01, ***P < 0.001, ****P < 0.001. [file 40478_2021_1216_MOESM3_ESM.pdf]

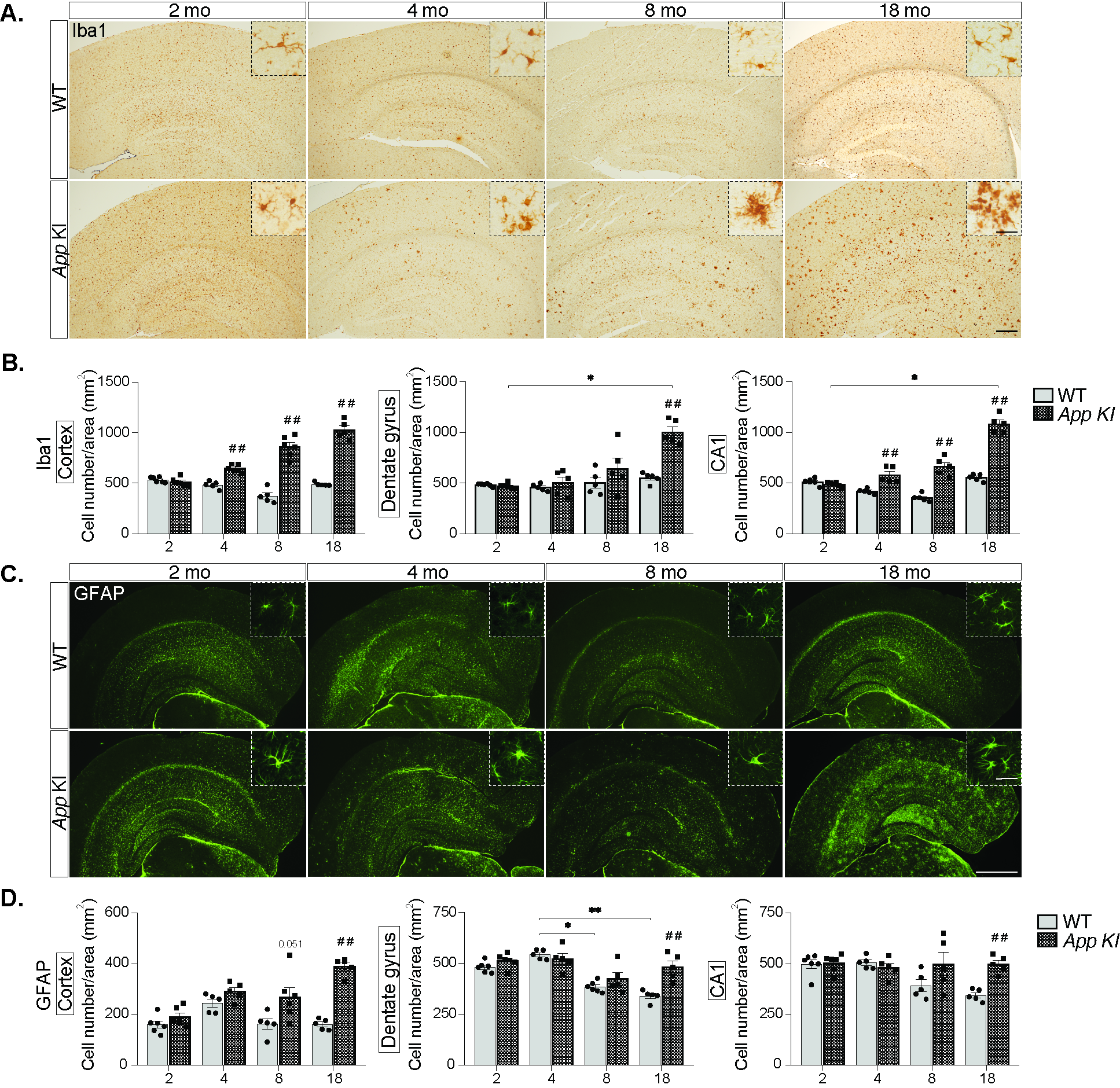

Supplement: Supplementary file 4 — Additional file 4: Fig. S4. (A, B) Sections stained for Iba1 show significantly higher number of Iba1-positive cells in App KI mice compared to WT mice starting at 4 months of age in cortex, DG and CA1. Cells were counted in three fields per section and two sections for each animal at 10x magnification. (C, D) Sections stained for GFAP show increased numbers of GFAP-positive cells in the cerebral cortex, DG and CA1 of 18 months old App KI mice compared to WT mice. Cells were counted in six fields per section and two sections per animal at 20x magnification. Cell numbers were normalized to area (mean ± SEM) (n = 5-6/group). Kruskal-Wallis one-way analysis of variance test with Dunn’s multiple comparison post hoc test *P < 0.05, **P < 0.01, ***P < 0.001; Mann-Whitney U test #P < 0.05, ##P < 0.01, ###P < 0.001). Scale bars = 30 and 300 μm. Iba1 = ionized calcium-binding adapter molecule 1, GFAP = glial fibrillary acidic protein, DG = dentate gyrus, CA1 = Cornu Ammonis 1. [file 40478_2021_1216_MOESM4_ESM.tif]

**A.****CORTEX**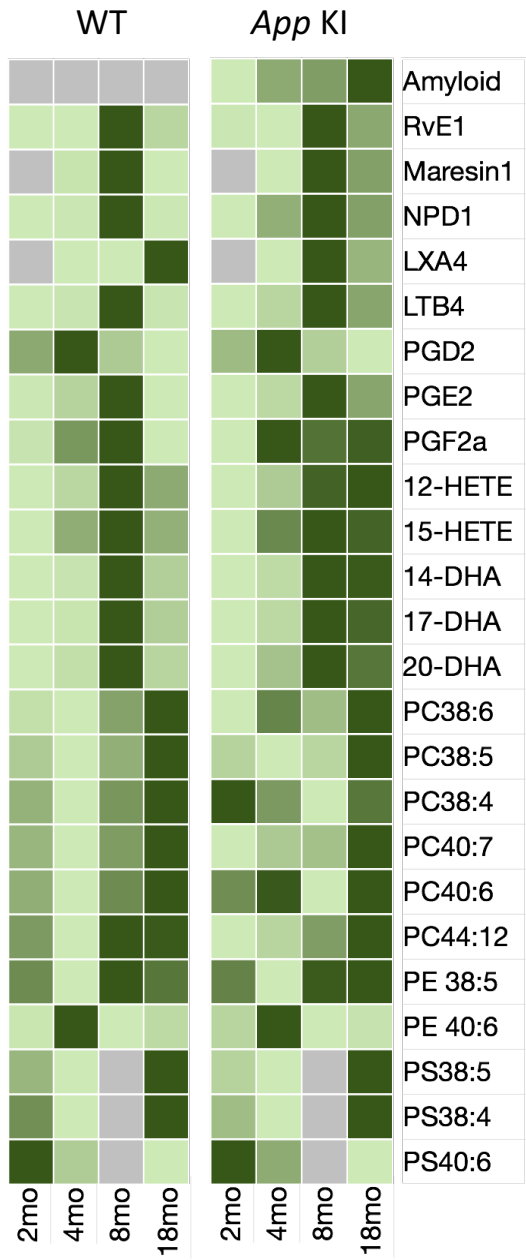**B.****HIPPOCAMPUS**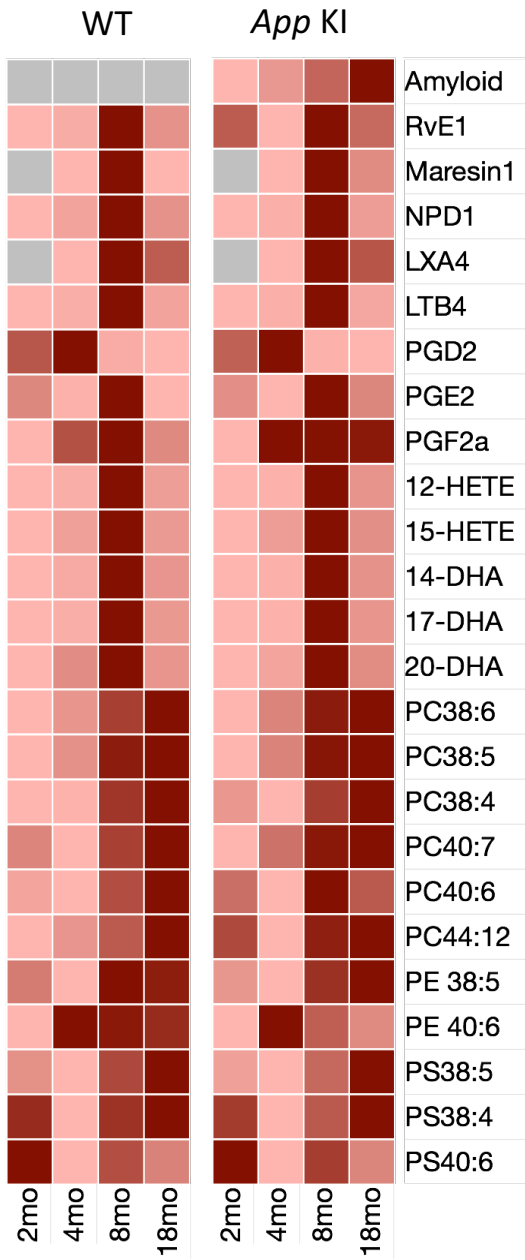

row max

row min

Supplement: Supplementary file 5 — Additional file 5: Fig. S5. Heat map analysis of bioactive LMs, AA- and DHA-containing phospholipids and amyloid levels in 2, 4, 8 and 18 months-old WT (n = 4-6) and App KI mice (n = 6-7). Rows represent the median values and columns represent different ages of WT and App KI mice. Grey colour represents undetected value. [file 40478_2021_1216_MOESM5_ESM.pdf]
